# Supplementary material for: Body Shape Phenotypes and Breast Cancer Risk: A Mendelian Randomization Analysis
Source: Cancers (Basel). 2023 Feb 17;15(4):1296. doi: 10.3390/cancers15041296 (PMC9954632; doi:10.3390/cancers15041296)
Supplement: Supplementary file 1 [file cancers-15-01296-s001.zip › Supplementary_Figures_Tables.pdf]

Supplementary Figures and Tables

Supplementary Figure S1 2

Supplementary Figure S2 3

Supplementary Figure S3 6

Supplementary Figure S4 7

Supplementary Table S1 8

Supplementary Table S2 9

Supplementary Table S3 10

Supplementary Table S4 11

References 12

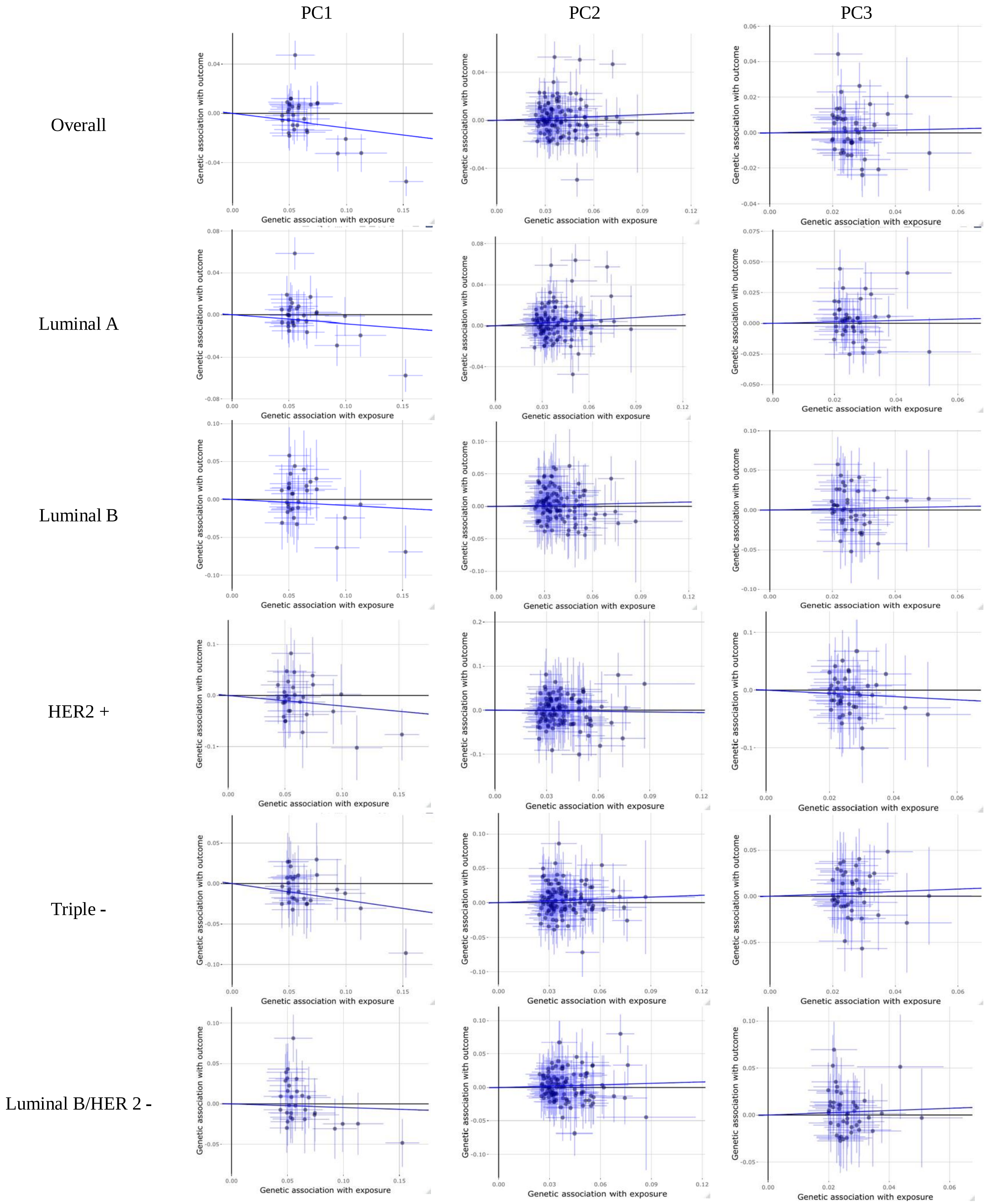

Supplementary Figure S1. Scatter plots of associations between body shapes and overall breast cancer and sub-types.

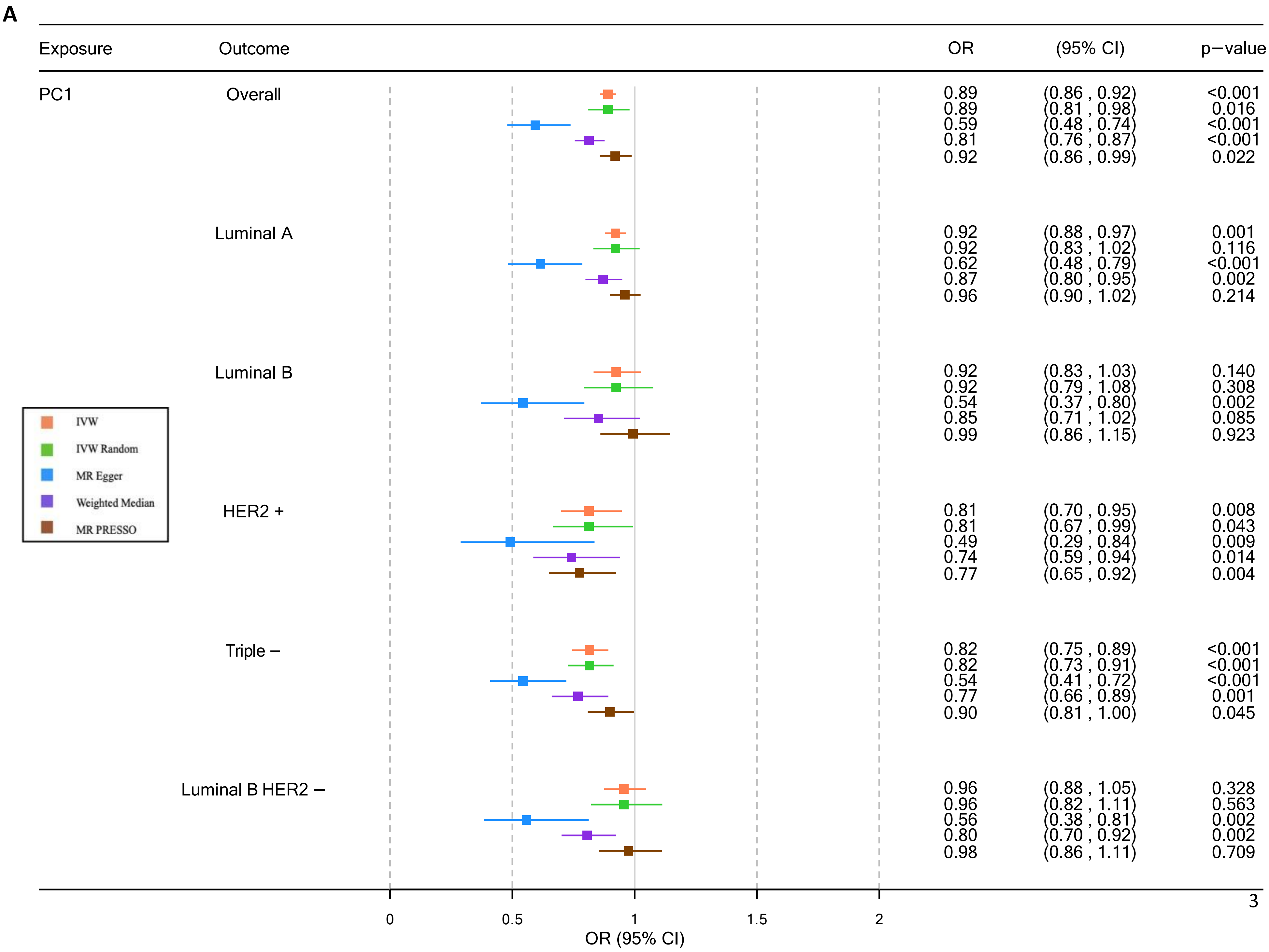

A
Exposure
Outcome
OR
(95% CI)
p−value
PC1
Overall
0.89 (0.86 , 0.92) <0.001
0.89 (0.81 , 0.98) 0.016
0.59 (0.48 , 0.74) <0.001
0.81 (0.76 , 0.87) <0.001
0.92 (0.86 , 0.99) 0.022
Luminal A
0.92 (0.88 , 0.97) 0.001
0.92 (0.83 , 1.02) 0.116
0.62 (0.48 , 0.79) <0.001
0.87 (0.80 , 0.95) 0.002
0.96 (0.90 , 1.02) 0.214
Luminal B
0.92 (0.83 , 1.03) 0.140
0.92 (0.79 , 1.08) 0.308
0.54 (0.37 , 0.80) 0.002
0.85 (0.71 , 1.02) 0.085
0.99 (0.86 , 1.15) 0.923
HER2 +
0.81 (0.70 , 0.95) 0.008
0.81 (0.67 , 0.99) 0.043
0.49 (0.29 , 0.84) 0.009
0.74 (0.59 , 0.94) 0.014
0.77 (0.65 , 0.92) 0.004
Triple −
0.82 (0.75 , 0.89) <0.001
0.82 (0.73 , 0.91) <0.001
0.54 (0.41 , 0.72) <0.001
0.77 (0.66 , 0.89) 0.001
0.90 (0.81 , 1.00) 0.045
Luminal B HER2 −
0.96 (0.88 , 1.05) 0.328
0.96 (0.82 , 1.11) 0.563
0.56 (0.38 , 0.81) 0.002
0.80 (0.70 , 0.92) 0.002
0.98 (0.86 , 1.11) 0.709
IVW
IVW Random
MR Egger
Weighted Median
MR PRESSO
0
0.5
1
1.5
2
OR (95% CI)

B

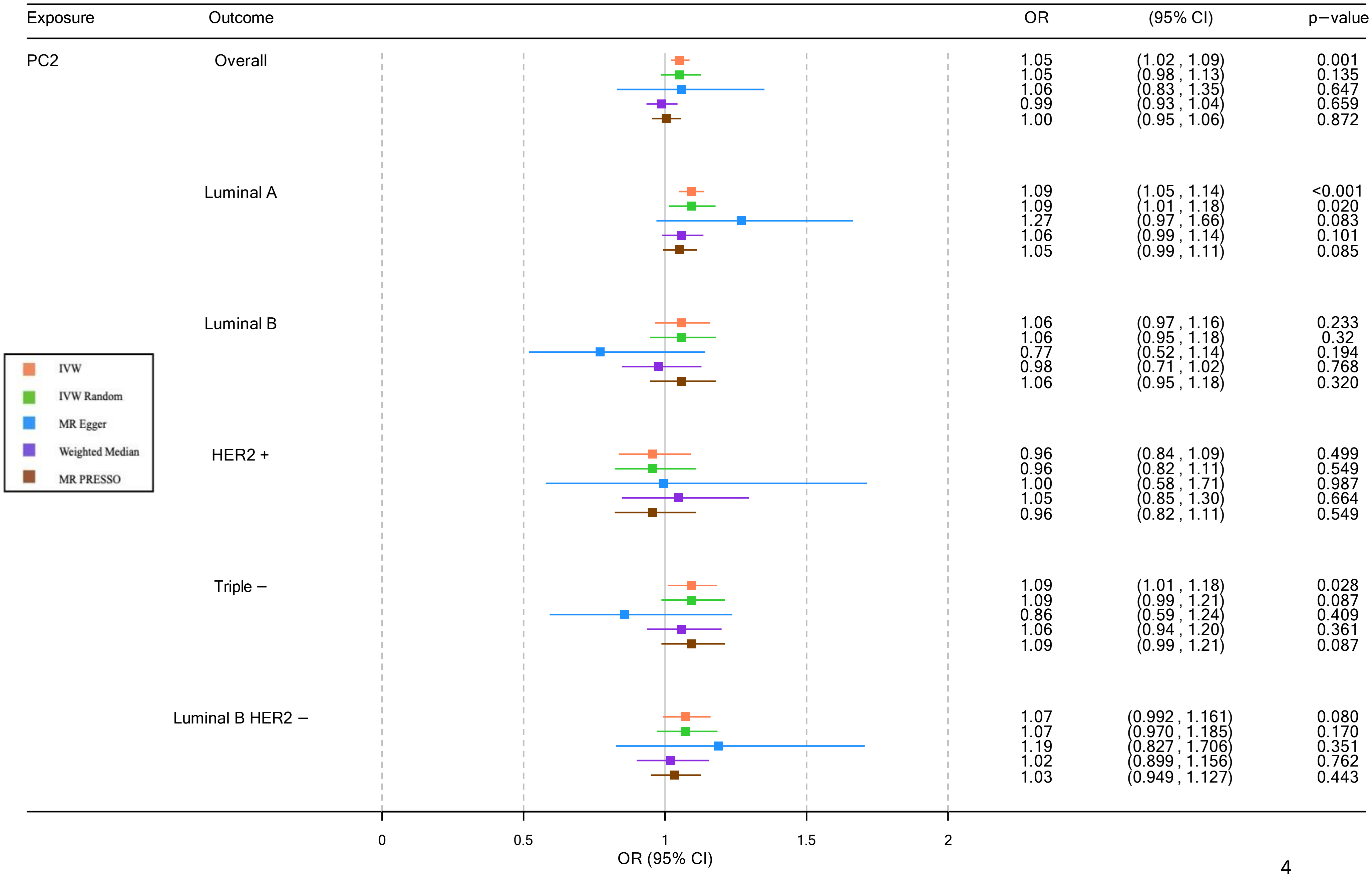
Exposure
Outcome
OR
(95% CI)
p−value
PC2
Overall
1.05 (1.02 , 1.09) 0.001
1.05 (0.98 , 1.13) 0.135
1.06 (0.83 , 1.35) 0.647
0.99 (0.93 , 1.04) 0.659
1.00 (0.95 , 1.06) 0.872
Luminal A
1.09 (1.05 , 1.14) <0.001
1.09 (1.01 , 1.18) 0.020
1.27 (0.97 , 1.66) 0.083
1.06 (0.99 , 1.14) 0.101
1.05 (0.99 , 1.11) 0.085
Luminal B
1.06 (0.97 , 1.16) 0.233
1.06 (0.95 , 1.18) 0.32
0.77 (0.52 , 1.14) 0.194
0.98 (0.71 , 1.02) 0.768
1.06 (0.95 , 1.18) 0.320
HER2 +
0.96 (0.84 , 1.09) 0.499
0.96 (0.82 , 1.11) 0.549
1.00 (0.58 , 1.71) 0.987
1.05 (0.85 , 1.30) 0.664
0.96 (0.82 , 1.11) 0.549
Triple −
1.09 (1.01 , 1.18) 0.028
1.09 (0.99 , 1.21) 0.087
0.86 (0.59 , 1.24) 0.409
1.06 (0.94 , 1.20) 0.361
1.09 (0.99 , 1.21) 0.087
Luminal B HER2 −
1.07 (0.992 , 1.161) 0.080
1.07 (0.970 , 1.185) 0.170
1.19 (0.827 , 1.706) 0.351
1.02 (0.899 , 1.156) 0.762
1.03 (0.949 , 1.127) 0.443
IVW
IVW Random
MR Egger
Weighted Median
MR PRESSO
0
0.5
1
1.5
2
OR (95% CI)

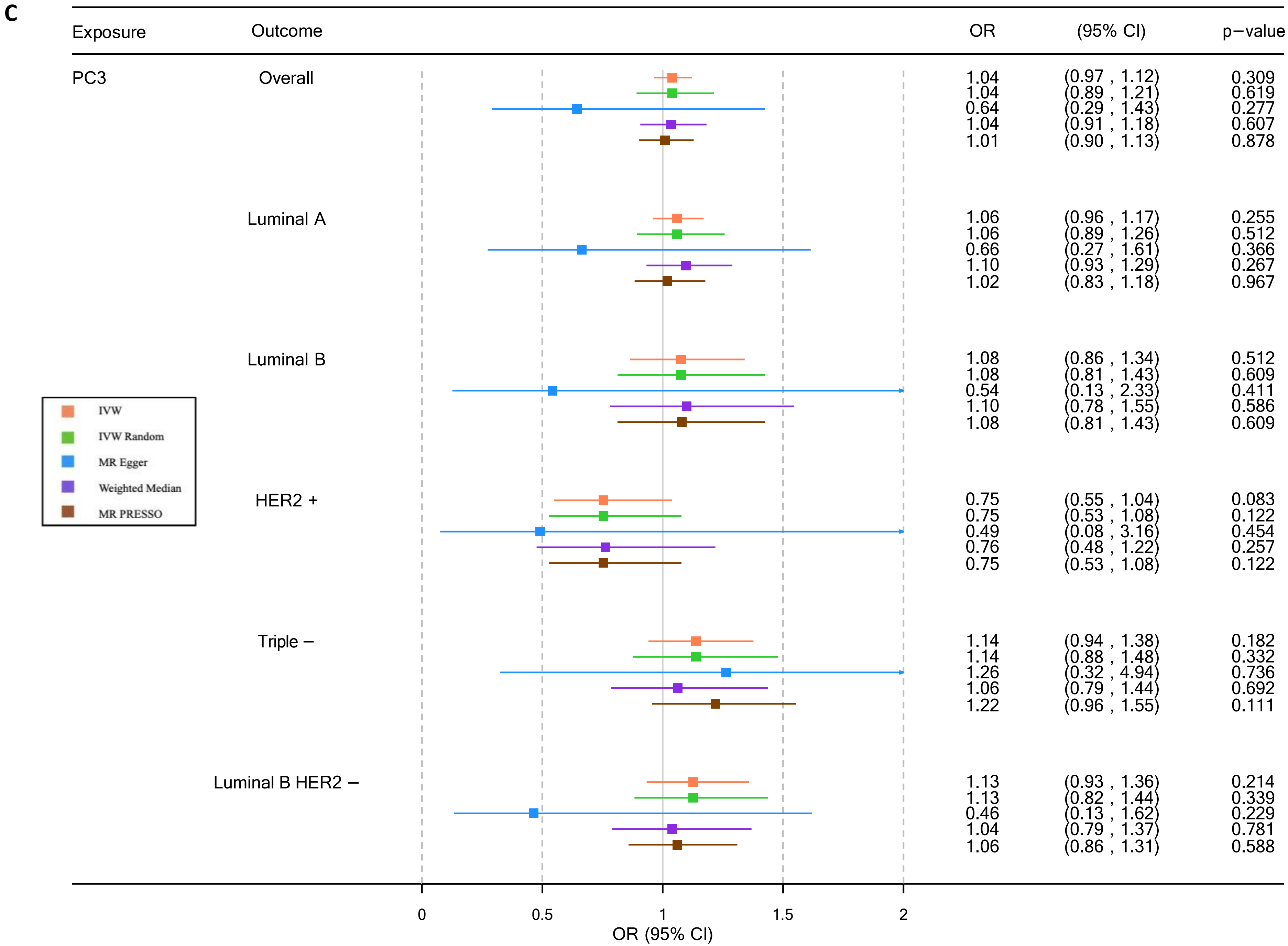

Supplementary Figure S2. Mendelian randomization estimates between body shape phenotype PC1 (A), PC2 (B) and PC3 (C) and overall and sub-types breast cancer risk using IVW (orange), IVW Random (green), MR Egger (blue), Weighted Median (violet) and MR PRESSO (brown) MR approaches.CI, confidence interval; OR, odds ratio.

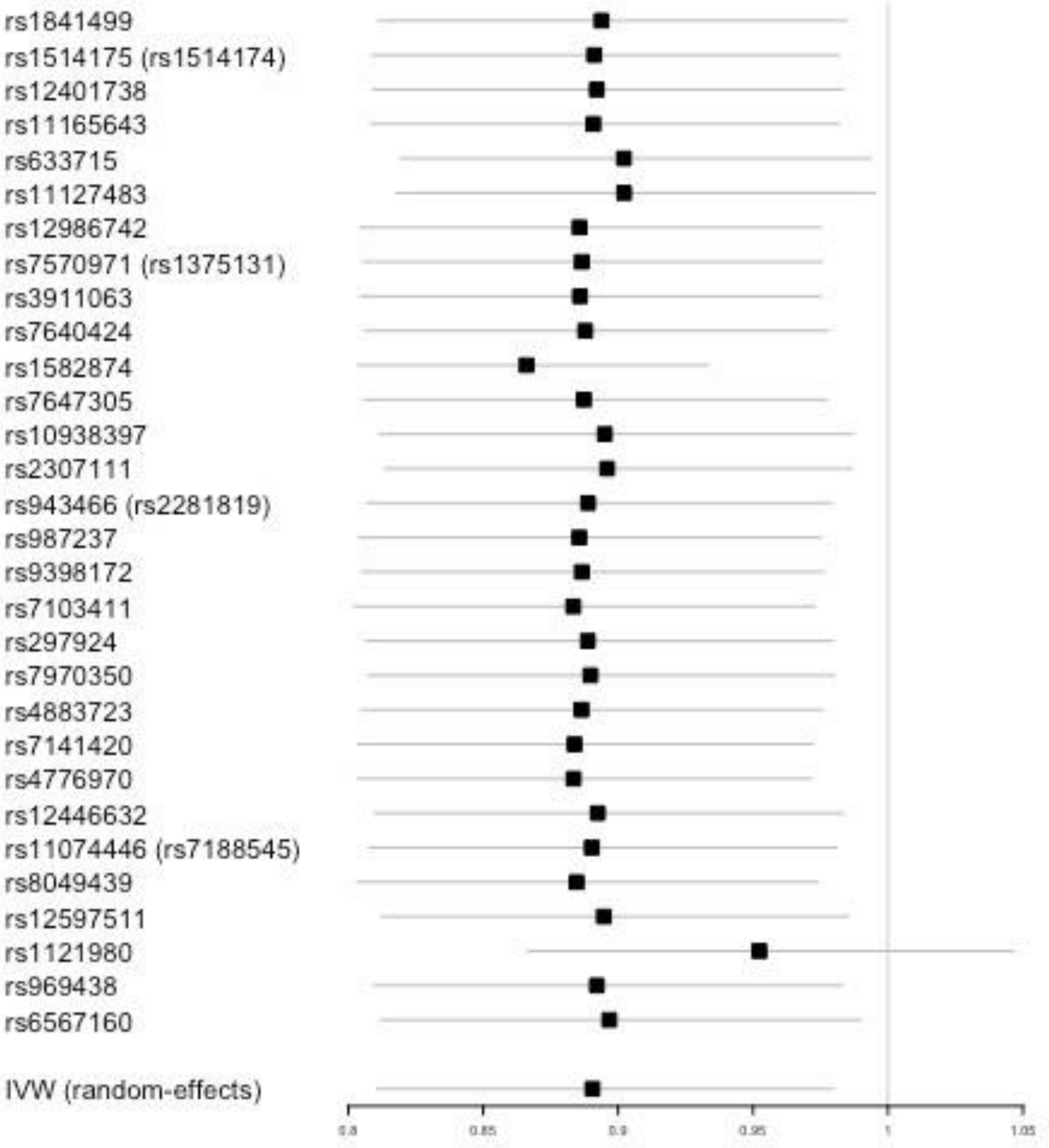

Supplementary Figure S3. Leave-one SNP-out plot for PC1 and overall breast cancer risk.

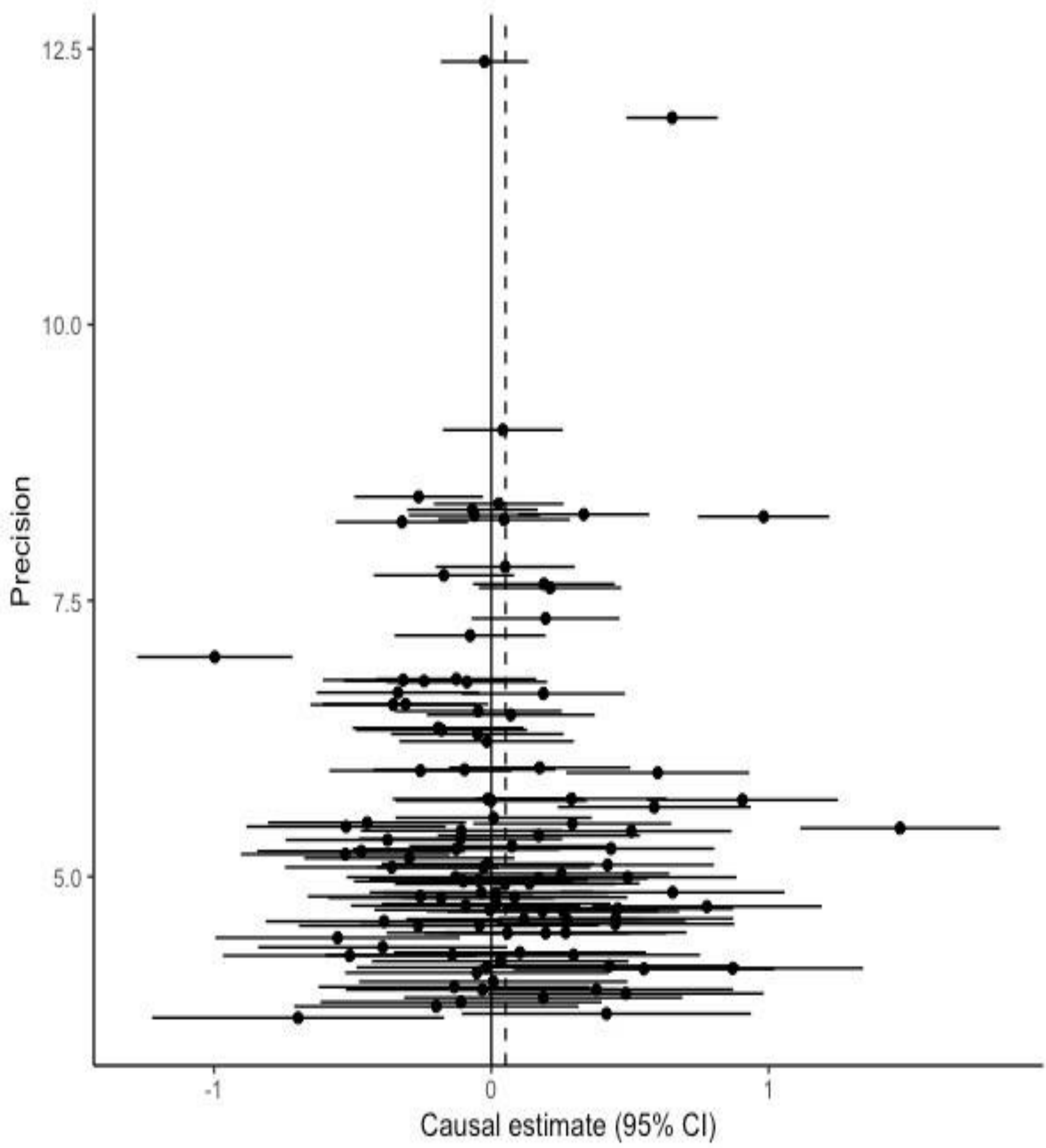

Supplementary Figure S4. Funnel plot for PC2 and overall breast cancer risk.

	PC1	PC2	PC3
**BMI**	0.473	-0.128	-0.284
**Height**	0.131	0.803	0.513
**Hip**	0.444	0.197	-0.399
**Waist**	0.488	-0.159	0.138
**Weight**	0.486	0.186	-0.056
**WHR**	0.297	-0.490	0.689
**Explained variance**	64.37%	18.46%	13.79%

Supplementary Table S1. Loadings and explained variance of the three PCs.[1]

Breast cancer GWAS sample size		
**Breast cancer**	**Overall**	**Sub-types**
**Cases**	133,384	106,278 (invasive cases)
**Controls**	113,789	91,477
**Tumor marker sub-types GWAS sample size**		
**Estrogen Receptor status**	**Negative**	16883
	**Positive**	69963
	**Unknown**	19432
**Progesterone Receptor status**	**Negative**	24260
	**Positive**	51546
	**Unknown**	30472
**HER2 status**	**Negative**	47645
	**Positive**	9502
	**Unknown**	49131
**Grade**	**1**	15566
	**2**	37532
	**3**	24360
	**Unknown**	28820
**Breast cancer sub-types description**		
Luminal A	ER+ and/or PR+, HER2-, grade 1 and 2	
Luminal B	ER+ and/or PR+, HER2+	
HER2 +	ER-, PR-, HER2+	
Triple -	ER-, PR-, HER2 -	
Luminal B/HER 2 -	ER+ and/or PR+, HER2-, grade 3	

Supplementary Table S2. Sample size for overall breast cancer and sub-types GWAS as well as according to tumor marker (Estrogen Receptor (negative, positive and unknown), Progesterone Receptor (negative, positive and unknown), HER2 (negative, positive and unknown), and grade (1, 2, 3 and unknown)). Finally, sub-types studied description regarding tumor markers.[2]

Outcome	Exposure	Heterogeneity			Egger intercept		
		Cochran's Q	df	p-value	Estimate	95% CI	p-value
**Overall**	**PC1**	199.5858	29	<0.001	0.029	0.014 , 0.043	<0.001
	**PC2**	515.5738	113	<0.001	0	-0.010 , 0.009	0.961
	**PC3**	180.1254	43	<0.001	0.013	-0.008 , 0.033	0.228
**Luminal A**	**PC1**	136.8719	29	<0.001	0.029	0.012 , 0.045	0.001
	**PC2**	368.3768	113	<0.001	-0.006	-0.017 , 0.004	0.256
	**PC3**	129.4055	43	<0.001	0.012	-0.011 , 0.036	0.293
**Luminal B**	**PC1**	60.9838	29	0.0005	0.038	0.012 , 0.063	0.003
	**PC2**	162.6251	113	0.0016	0.013	-0.003 , 0.028	0.1
	**PC3**	70.7185	43	0.0049	0.018	-0.02 , 0.056	0.348
**HER2 +**	**PC1**	49.9252	29	0.0092	0.036	0.001 , 0.071	0.046
	**PC2**	143.6335	113	0.0273	-0.002	-0.023 , 0.02	0.876
	**PC3**	54.0215	43	0.1209	0.011	-0.037 , 0.06	0.644
**Triple -**	**PC1**	45.7765	29	0.0247	0.029	0.01 , 0.048	0.003
	**PC2**	187.1135	113	<0.001	0.01	-0.004 , 0.025	0.175
	**PC3**	81.4148	43	0.0004	-0.003	-0.038 , 0.033	0.878
**Luminal B/ HER2 -**	**PC1**	82.7505	29	<0.001	0.038	0.013 , 0.063	0.003
	**PC2**	183.9729	113	<0.001	-0.004	-0.018 , 0.01	0.565
	**PC3**	72.6526	43	0.0031	0.023	-0.009 , 0.056	0.156

Supplementary Table S3. Heterogeneity test and Egger intercept results for body shape phenotypes and overall breast cancer risk and sub-types.

Breast Cancer	PC1	PC2	PC3
**Overall**	rs1582874<br>rs1121980	rs11205303<br>rs724016<br>rs6569648<br>rs11049611<br>rs3764419	rs9425301<br>rs6726261<br>rs9838625<br>rs272885<br>rs1936809
	ρ = 0.130	ρ = 0.013	ρ = 0.123
**Luminal A**	rs1582874<br>rs1121980	rs11205303<br>rs724016<br>rs6569648<br>rs11049611<br>rs1182199	rs9838625<br>rs272885<br>rs1516883
	ρ = 0.053	ρ = 0.020	ρ = 0.038
**Luminal B**	rs3911063<br>rs1121980	-	-
	ρ = 0.039		
**HER2 +**	rs1582874	-	-
	ρ = 0.692		
**Triple -**	rs1121980	rs6569648<br>rs11049611	rs9425301
	ρ = 0.007	ρ = 0.769	ρ = 0.545
**Luminal B/HER2 -**	rs1582874<br>rs1121980	rs1415288<br>rs724016<br>rs11049611	rs9838625
	ρ = 0.334	ρ = 0.204	ρ = 0.266

Supplementary Table S4. Genetic variants considered as outliers for overall and sub-types breast cancer and each body shape phenotype. We also provide P-distortion (ρ) for each testperformed.

References

1. Ried JS, Jeff JM, Chu AY, Bragg-Gresham JL, Van Dongen J, Huffman JE, et al. A principal component meta-analysis on multiple anthropometric traits identifies novel loci for body shape. Nat Commun. 2016;7.
2. Zhang H, Ahearn TU, Lecarpentier J, Barnes D, Beesley J, Qi G, et al. Genome-wide association study identifies 32 novel breast cancer susceptibility loci from overall and subtype-specific analyses. Nat Genet. 2020;52(6).
