# Supplementary material for: Body Shape Phenotypes and Breast Cancer Risk: A Mendelian Randomization Analysis
Source: Cancers (Basel). 2023 Feb 17;15(4):1296. doi: 10.3390/cancers15041296 (PMC9954632; doi:10.3390/cancers15041296)
Supplement: Supplementary file 1 [file cancers-15-01296-s001.zip › Supplementary_File_S3.pdf]

Supplementary File 1: Genetic variants and associated traits in previous GWAS ($p < 5x10^{-8}$)

PC1	
SNP (lead SNP)	Trait
rs1841499	Age at menarche<br>Alcohol intake versus 10 years previously<br>Ambidextrous<br>Basal metabolic rate<br>Body fat percentage<br>BMI<br>Fluid intelligence score<br>Hayfever, allergic rhinitis or eczema<br>Hip circumference<br>Impedance<br>Long-standing illness, disability or infirmity<br>Medication for cholesterol, blood pressure or diabetes: none of the above<br>No blood clot, bronchitis, emphysema, asthma, rhinitis, eczema or allergy diagnosed by doctor<br>Number of self-reported non-cancer illnesses<br>umber of treatments or medications taken<br>Obesity class 1<br>Obesity class 2<br>Obesity class 3<br>Overweight<br>Pulse rate<br>Qualifications: college or university degree<br>Qualifications: none<br>Qualifications: other professional qualifications<br>Relative age of first facial hair<br>Seen doctor for nerves, anxiety, tension or depression<br>Sodium in urine

	Types of transport used, excluding work: public transport<br>Waist circumference<br>Weight<br>Whole body fat mass<br>Whole body fat-free mass<br>Whole body water mass<br>Years of educational attainment
rs1514175 (rs1514174)	Age at menarche<br>Basal metabolic rate<br>BMI<br>Childhood BMI<br>Childhood obesity<br>Ever smoked<br>Extreme obesity body mass index<br>Extreme obesity with early age of onset<br>Hip circumference<br>Impedance<br>Obesity class 1<br>Obesity with early age of onset age 2<br>Overweight<br>Past tobacco smoking<br>Relative age of first facial hair<br>Relative age voice broke<br>Waist circumference<br>Weight<br>Whole body fat mass<br>Whole body fat-free mass<br>Whole body water mass
	Basal metabolic rate<br>BMI<br>Body fat percentage<br>Height

rs12401738	Hip circumference<br>Impedance<br>Waist circumference<br>Weight<br>Whole body fat mass<br>Whole body fat-free mass<br>Whole body water mass<br>Years of educational attainment
rs11165643	Basal metabolic rate<br>BMI<br>Body fat percentage<br>Hip circumference<br>Treatment with olmesartan<br>Waist circumference<br>Weight<br>Whole body fat mass<br>Whole body fat-free mass<br>Whole body water mass
rs633715	Age at menarche<br>Basal metabolic rate<br>BMI<br>Body fat percentage<br>Childhood BMI<br>Childhood obesity<br>Hip circumference<br>Impedance<br>Menarche<br>Menarche age at onset<br>Obesity<br>Obesity class 1<br>Obesity class 2<br>Obesity class 3

	Obesity with early age of onset age 2<br>Overweight<br>Relative age of first facial hair<br>Waist circumference<br>Waist hip ratio<br>Weight<br>Whole body fat mass<br>Whole body fat-free mass<br>Whole body water mass
rs11127483	Age at menarche<br>Basal metabolic rate<br>BMI<br>Body fat percentage<br>Childhood BMI<br>Childhood obesity<br>Diastolic blood pressure<br>Heel bone mineral density<br>Hip circumference<br>Impedance<br>Obesity class 1<br>Obesity class 2<br>Obesity class 3<br>Obesity with early age of onset age 2<br>Overweight<br>Past tobacco smoking<br>Relative age of first facial hair<br>Systolic blood pressure<br>Waist circumference<br>Weight<br>Whole body fat mass<br>Whole body fat-free mass<br>Whole body water mass

rs12986742	Basal metabolic rate<br>BMI<br>Body fat percentage<br>Getting up in morning<br>Hip circumference<br>Impedance<br>Waist circumference<br>Weight<br>Whole body fat mass<br>Whole body fat-free mass<br>Whole body water mass
rs7570971 (rs1375131)	Blood metabolite levels<br>BMI<br>Body fat percentage<br>Cholesterol<br>Forced expiratory volume in 1 sec<br>Forced vital capacity<br>Hand grip strength<br>Height<br>Hip circumference<br>LDL<br>Low density lipoprotein<br>Monocyte percentage of white cells<br>Parkinsons disease<br>Sleep duration<br>Total cholesterol<br>Type II diabetes<br>Waist circumference<br>Weight<br>Whole body fat mass
	Basal metabolic rate

rs3911063	BMI<br>Body fat percentage<br>Ever smoked<br>Hip circumference<br>Impedance<br>Past tobacco smoking<br>Qualifications: A levels or as levels or equivalent<br>Qualifications: college or university degree<br>Qualifications: other professional qualifications<br>Smoking status: previous<br>Waist circumference<br>Weight<br>Whole body fat mass<br>Whole body fat-free mass<br>Whole body water mass<br>Years of educational attainment
rs7640424	BMI<br>Hip circumference<br>Impedance<br>Qualifications: A levels or as levels or equivalent<br>Years of educational attainment
	Allergic disease<br>Basal metabolic rate<br>Birth weight of first child<br>Body fat percentage<br>Ever had prostate specific antigen test<br>Forced expiratory volume in 1 sec<br>Forced vital capacity<br>Hair or balding pattern<br>Hand grip strength<br>Hayfever, allergic rhinitis or eczema<br>Heel bone mineral density

rs1582874	Height<br>Hip circumference<br>Lymphocyte percentage of white cells<br>Mean corpuscular hemoglobin<br>Mean corpuscular volume<br>Myopia<br>Neutrophil percentage of white cells<br>No blood clot, bronchitis, emphysema, asthma, rhinitis, eczema or allergy diagnosed by doctor<br>Potassium in urine<br>Pulse rate<br>Red blood cell count<br>Relative age of first facial hair<br>Self-reported enlarged prostate<br>Waist circumference<br>Weight<br>Whole body fat mass<br>Whole body fat-free mass<br>Whole body water mass
rs7647305	Basal metabolic rate<br>BMI<br>Hip circumference<br>Impedance<br>Obesity class 1<br>Obesity class 2<br>Overweight<br>Waist circumference<br>Weight<br>Whole body fat mass<br>Whole body fat-free mass<br>Whole body water mass
	Age at menarche<br>Basal metabolic rate

rs10938397	BMI<br>Body fat percentage<br>Childhood BMI<br>Childhood obesity<br>Extreme obesity with early age of onset<br>Hip circumference<br>Impedance<br>log Fast insulin<br>Menarche age at onset<br>Obesity<br>Obesity class 1<br>Obesity class 2<br>Obesity class 3<br>Obesity with early age of onset age 2<br>Overweight<br>Relative age voice broke<br>Waist circumference<br>Waist hip ratio<br>Weight<br>Whole body fat mass<br>Whole body fat-free mass<br>Whole body water mass
	Basal metabolic rate<br>BMI<br>Body fat percentage<br>Diabetes diagnosed by doctor<br>Diastolic blood pressure<br>Hip circumference<br>Impedance<br>LDL cholesterol<br>Low density lipoprotein<br>Obesity

rs2307111	Obesity class 1<br>Obesity class 2<br>Overweight<br>Pain type experienced in last month: headache<br>Platelet count<br>Pulse rate<br>Self-reported diabetes<br>Self-reported high cholesterol<br>Snoring<br>Total cholesterol<br>Usual walking pace<br>Waist circumference<br>Weight<br>Whole body fat mass<br>Whole body fat-free mass<br>Whole body water mass
rs943466 (rs2281819)	BMI<br>Body fat percentage<br>Hip circumference<br>Pack years adult smoking as proportion of life span exposed to smoking<br>Platelet count<br>Plateletcrit<br>Pulse rate<br>Waist circumference<br>Whole body fat mass
	Adiposity<br>Age at menarche<br>Basal metabolic rate<br>BMI<br>Body fat percentage<br>Chilhood BMI<br>Diabetes diagnosed by doctor

rs987237	Extreme obesity with early age of onset<br>Getting up in morning<br>Hip circumference<br>Impedance<br>Medication for cholesterol, blood pressure or diabetes: blood pressure medication<br>Medication for cholesterol, blood pressure or diabetes: none of the above<br>Morning or evening person<br>Obesity class 1<br>Obesity class 2<br>Obesity class 3<br>Overweight<br>Self-reported diabetes<br>Treatment with metformin<br>Waist circumference<br>Waist hip ratio<br>Weight<br>Whole body fat mass<br>Whole body fat-free mass<br>Whole body water mass
rs9398172	Age at menarche<br>Basal metabolic rate<br>BMI<br>Fluid intelligence score<br>Height<br>High light scatter percetage of red cells<br>Hip circumference<br>Impedance<br>Intelligence<br>Intracranial volume<br>Mean time to correctly identify matches<br>Number of days or week of vigorous physical activity 10+ minutes<br>Peak expiratory flow

	Qualifications: college or university degree<br>Qualifications: none<br>Relative age of first facial hair<br>Schizophrenia<br>Waist circumference<br>Weight<br>Whole body fat mass<br>Whole body fat-free mass<br>Whole body water mass
rs4536164 (rs4514364)	Age at menarche<br>Basal metabolic rate<br>BMI<br>Body fat percentage<br>Hip circumference<br>Impedance<br>Lumbar spine bone mineral density in females<br>Obesity class 1<br>Overweight<br>Survival in pancreatic cancer<br>Waist circumference<br>Weight<br>Whole body fat mass<br>Whole body fat-free mass<br>Whole body water mass
	Age at menarche<br>Basal metabolic rate<br>BMI<br>Body fat percentage<br>Chilhood BMI<br>Coronary artery disease<br>Ever smoked<br>Falls in the last year

rs7103411	Forced vital capacity<br>Hip circumference<br>Impedance<br>Menarche age at onset<br>Obesity class 1<br>Obesity class 2<br>Overweight<br>Past tobacco smoking<br>Relative age of first facial hair<br>Relative age voice broke<br>Snoring<br>Waist circumference<br>Waist hip ratio<br>Weight<br>Whole body fat mass<br>Whole body fat-free mass<br>Whole body water mass
rs297924	Age at menarche<br>Basal metabolic rate<br>BMI<br>Body fat percentage<br>Childhood BMI<br>Chilhood obesity<br>Hip circumference<br>Home area population density: Scotland large urban area<br>Impedance<br>Obesity<br>Obesity class 1<br>Obesity class 2<br>Obesity with early age of onset age 2<br>Waist circumference<br>Weight

	Whole body fat mass<br>Whole body fat-free mass<br>Whole body water mass
rs7970350	Alcohol intake frequency<br>Basal metabolic rate<br>Birth weight<br>Birth weight of first child<br>Birthlenght<br>BMI in males less than or equal to 50 years of age<br>Diabetes diagnosed by doctor<br>Forced expiratory volume in 1 sec<br>Forced vital capacity<br>Hand grip strength<br>Height<br>Hip circumference<br>Impedance<br>Infant head circumference<br>Infant length<br>Maternal effects on offspring birthweight<br>Number of self-reported non-cancer illnesses<br>Overall health rating<br>Peak expiratory flow<br>Pulse rate<br>Self-reported diabetes<br>Self-reported high cholesterol<br>Systolic blood pressure<br>Treatment with metformin<br>Waist circumference<br>Weight<br>Whole body fat mass<br>Whole body fat-free mass<br>Whole body water mass

rs4883723	Basal metabolic rate<br>BMI<br>Body fat percentage<br>Childhood BMI<br>Childhood obesity<br>Hip circumference<br>Leg impedance<br>Obesity with early age of onset age 2<br>Waist circumference<br>Weight<br>Whole body fat mass<br>Whole body fat-free mass<br>Whole body water mass
rs7141420	Basal metabolic rate<br>BMI<br>Body fat percentage<br>Hip circumference<br>Impedance<br>Obesity<br>Obesity class 1<br>Obesity class 2<br>Overweight<br>Sodium in urine<br>Waist circumference<br>Waist hip ratio<br>Weight<br>Whole body fat mass<br>Whole body fat-free mass<br>Whole body water mass
	Age at menarche<br>Basal metabolic rate

rs4776970	BMI<br>Body fat percentage<br>Diastolic blood pressure<br>Hip circumference<br>Impedance<br>Obesity class 1<br>Obesity class 2<br>Overweight<br>Past tobacco smoking<br>Vascular or heart problems diagnosed by doctor: high blood pressure<br>Vascular or heart problems diagnosed by doctor: none of the above<br>Waist circumference<br>Waist hip ratio<br>Weight<br>Whole body fat mass<br>Whole body fat-free mass<br>Whole body water mass
rs12446632	Age at menarche<br>Basal metabolic rate<br>BMI<br>Childhood BMI<br>Hand grip strength<br>Heel bone mineral density<br>High density lipoprotein<br>Hip circumference<br>Impedance<br>Menarche age at onset<br>Obesity<br>Obesity class 1<br>Obesity class 2<br>Overweight<br>Waist circumference

	Weight<br>Whole body fat mass<br>Whole body fat-free mass<br>Whole body water mass
rs11074446 (rs7188545)	Basal metabolic rate<br>BMI<br>Body fat percentage<br>Hip circumference<br>Impedance<br>Overweight<br>Waist circumference<br>Whole body fat mass<br>Whole body fat-free mass<br>Whole body water mass
rs8049439	Alcohol intake frequency<br>Basal metabolic rate<br>BMI<br>Body fat percentage<br>Crohns disease<br>Educational attainment<br>Fluid intelligence score<br>Gene expression<br>Hand grip strength<br>Hip circumference<br>Inflammatory bowel disease<br>Inflammatory bowel disease early onset<br>Intelligence<br>Intelligence multi trait analysis<br>Leg impedance<br>Mean corpuscular volume<br>Nonsyndromic striar distensae stretch marks<br>Obesity class 1

	Obesity class 2<br>Overweight<br>Qualifications: college or university degree<br>Qualifications: none<br>Red blood cell count<br>Suffer from nerves<br>Types of physical activity in last 4 weeks: light diy<br>Usual walking pace<br>Waist circumference<br>Waist hip ratio<br>Weight<br>Whole body fat mass<br>Whole body fat-free mass<br>Whole body water mass<br>Years of educational attainment
rs12597511	Alcohol intake frequency<br>Basal metabolic rate<br>BMI<br>Body fat percentage<br>Diastolic blood pressure<br>Dose change in phenprocoumon warfarin in mgweek per allele<br>Forced expiratory volume in 1 sec<br>Forced vital capacity<br>Hip circumference<br>Illnessess of siblings: high blood pressure<br>Illnessess of siblings: none of the above<br>Impedance<br>Lymphocyte count<br>Mean corpuscular hemoglobin<br>Mean corpuscular volume<br>Nervous feelings<br>Number of incorrect marches in round

	Overall health rating<br>Self-reported hypertension<br>Snoring<br>Time spent watching tv<br>Types of physical activity in last 4 weeks: walking for pleasure<br>Usual walking pace<br>Vascular or heart problems diagnosed by doctor: high blood pressure<br>Vascular or heart problems diagnosed by doctor: none of the above<br>Waist circumference<br>Weight<br>Whole body fat mass<br>Whole body fat-free mass<br>Whole body water mass
	Age at menarche<br>Alcohol intake frequency<br>Average weekly beer plus cider intake<br>Average weekly red wine intake<br>Average weekly spirits intake<br>Basal metabolic rate<br>BMI<br>Body fat percentage<br>Childhood BMI<br>Childhood obesity<br>Creatinine in urine<br>Cystatin C in serum<br>Diabetes diagnosed by doctor<br>Extreme obesity with early age of onset<br>Getting up in morning<br>HbA1c<br>HDL cholesterol<br>Heel bone mineral density<br>High density lipoprotein

rs1121980	Hip circumference<br>Illnesses of father: diabetes<br>Illnesses of mother: diabetes<br>Illnessess of siblings: high blood pressure<br>Impedance<br>log Fast insulin<br>log Leptin<br>Medication for cholesterol, blood pressure or diabetes: blood pressure medication<br>Medication for cholesterol, blood pressure or diabetes: none of the above<br>Morning or evening person<br>Number of treatments or medications taken<br>Obesity<br>Obesity class 1<br>Obesity class 2<br>Obesity class 3<br>Obesity early onset extreme<br>Obesity with early age of onset age 2<br>Overall health rating<br>Overweight<br>Relative age of first facial hair<br>Relative age voice broke<br>Self-reported breast cancer<br>Self-reported diabetes<br>Self-reported hypertension<br>Sleep duration<br>Snoring<br>Sodium in urine<br>Treatment with bendroflumethiazide<br>Treatment with blood pressure medication<br>Treatment with gliclazide<br>Treatment with metformin<br>Triglycerides

	Type II diabetes<br>Type II diabetes combined control dataset<br>Usual walking pace<br>Vascular or heart problems diagnosed by doctor: high blood pressure<br>Vascular or heart problems diagnosed by doctor: none of the above<br>Waist circumference<br>Waist hip ratio<br>Weight<br>Whole body fat mass<br>Whole body fat-free mass<br>Whole body water mass
rs969438	BMI<br>Obesity class 1<br>Trunk fat percentage<br>Waist circumference<br>Weight
	Alcohol intake frequency<br>Antipsychotic drug induced weight gain<br>Basal metabolic rate<br>BMI<br>Body fat percentage<br>Childhood BMI<br>Childhood obesity<br>Coronary artery disease<br>Diabetes diagnosed by doctor<br>Fat body mass<br>Forced expiratory volume in 1 sec<br>Hand grip strength<br>HDL cholesterol<br>Height<br>High density lipoprotein<br>Hip circumference

rs6567160	Impedance<br>Myocardial infarction<br>Nervous feelings<br>Obesity class 1<br>Obesity class 2<br>Obesity with early age of onset age 2<br>Overweight<br>Self-reported diabetes<br>Suffer from nerves<br>Treatment with levothyroxine sodium<br>Type II diabetes<br>Vascular or heart problems diagnosed by doctor: high blood pressure<br>Vascular or heart problems diagnosed by doctor: none of the above<br>Waist circumference<br>Waist hip ratio<br>Weight<br>Whole body fat mass<br>Whole body fat-free mass<br>Whole body water mass<br>Worrier or anxious feelings

PC2	
SNP (lead SNP)	Trait
rs2284746	BMI<br>Forced vital capacity<br>Gene expression<br>Height<br>Hematocrit<br>Hip circumference<br>Impedance<br>Pulmonary function<br>Waist circumference<br>Whole body fat-free mass<br>Whole body water mass
rs2903545	Hand grip strength<br>Height<br>Hip circumference<br>Sum eosinophil basophil counts<br>Trunk fat-free mass<br>Whole body fat-free mass<br>Whole body water mass
rs11209376	Creatinine in urine<br>Diastolic blood pressure<br>Forced expiratory volume in 1 sec<br>Forced vital capacity<br>Hand grip strength<br>Heel bone mineral density<br>Height<br>Hip circumference<br>Potassium in urine<br>Weight

	Whole body fat-free mass<br>Whole body water mass
rs17391694	Alcohol intake frequency<br>Basal metabolic rate<br>BMI<br>Creatinine in urine<br>Crohns disease<br>Forced vital capacity<br>Height<br>Hip circumference<br>Impedance<br>Leg fat percentage<br>Lung cancer<br>Mean corpuscular hemoglobin concentration<br>Time spent watching tv<br>Waist circumference<br>Weight<br>Whole body fat-free mass<br>Whole body water mass
rs7536458	Basal metabolic rate<br>Forced expiratory volume in 1 sec<br>Hair or balding pattern<br>Height<br>Hip circumference<br>Infant length<br>Leg impedance<br>Trunk fat-free mass<br>Waist circumference<br>Waist hip ratio<br>Whole body fat-free mass<br>Whole body water mass

rs11205303	Basal metabolic rate
	Body fat percentage
	Breast cancer
	Coronary artery disease
	Forced expiratory volume in 1 sec
	Forced vital capacity
	Forced vital capacity
	Granulocyte percentage of myeloid white cells
	Heel bone mineral density
	Height
	Hip circumference
	Hip circumference
	Impedance
	Infant length
	Trunk fat mass
	Trunk fat percentage
	Waist circumference
	Weight
	Whole body fat mass
	Whole body fat-free mass
	Whole body water mass
rs1752388 (rs2298265)	Height
rs905938	Basal metabolic rate
	Birth length
	Birth weight
	BMI
	Eosinophil percentage of granulocytes
	Hayfever, allergic rhinitis or eczema
	Height
	Hip circumference
	Impedance

	Neutrophil percentage of granulocytes<br>Sum eosinophil basophil counts<br>Trunk fat-free mass<br>Waist circumference<br>Waist hip ratio<br>Weight<br>Whole body fat-free mass<br>Whole body water mass
rs11584955	Basal metabolic rate<br>Forced expiratory volume in 1 sec<br>Forced vital capacity<br>Hair or balding pattern<br>Heel bone mineral density<br>Height<br>Hematocrit<br>Hemoglobin concentration<br>Hip circumference<br>Mean platelet volum<br>Platelet distribution width<br>Red blood cell count<br>Weight<br>Whole body fat-free mass<br>Whole body water mass
rs1014719	Basal metabolic rate<br>Forced expiratory volume in 1 sec<br>Forced vital capacity<br>Hand grip strength<br>Height<br>Impedance<br>Weight<br>Whole body fat-free mass

	Whole body water mass
rs1046934	Basal metabolic rate<br>Forced expiratory volume in 1 sec<br>Forced vital capacity<br>Gene expression<br>Hand grip strength<br>Height<br>Hip circumference<br>Impedance<br>Myopia<br>Waist circumference<br>Weight<br>Whole body fat-free mass<br>Whole body water mass
rs1415288	Adiponectin levels<br>Body fat percentage<br>Coxarthrosis<br>Falls in the last year<br>Femoral hernia<br>Height<br>Hip circumference<br>Impedance<br>Inguinal hernia<br>Trunk fat percentage<br>Type II diabetes<br>Umbilical hernia<br>Ventral hernia<br>Waist circumference<br>Waist hip ratio<br>Weight<br>Whole body fat mass

rs12093031 (rs10799424)	Forced expiratory volume in 1 sec<br>Forced vital capacity<br>Hand grip strength<br>Height<br>Hip circumference<br>Impedance<br>Trunk fat-free mass<br>Waist circumference
rs2278483	BMI<br>Body fat percentage<br>Childhood BMI<br>Forced vital capacity<br>Hand grip strength<br>Height<br>Hip circumference<br>Trunk fat-free mass<br>Waist circumference
rs2289195	Basal metabolic rate<br>Forced expiratory volume in 1 sec<br>Forced vital capacity<br>Hand grip strength<br>Height<br>Leg impedance<br>Waist circumference<br>Whole body fat-free mass<br>Whole body water mass
	Basal metabolic rate<br>Forced expiratory volume in 1 sec<br>Height

rs1545552	Hip circumference<br>Hip circumference<br>Weight<br>Whole body fat-free mass<br>Whole body water mass
rs6740539	Height
rs3791675	Basal metabolic rate<br>Forced expiratory volume in 1 sec<br>Forced vital capacity<br>Height<br>Hip circumference<br>Impedance<br>Inguinal hernia<br>Mononeuropathies of upper limb<br>Peak expiratory flow<br>Waist circumference<br>Weight<br>Whole body fat mass<br>Whole body fat-free mass<br>Whole body water mass
rs1913671	Getting up in morning<br>Height<br>Hip circumference<br>Trunk fat-free mass<br>Waist circumference<br>Whole body fat-free mass
	Basal metabolic rate<br>Height<br>Hip circumference

rs1478576	Impedance<br>Weight<br>Whole body fat mass<br>Whole body fat-free mass<br>Whole body water mass
rs3103267	Basal metabolic rate<br>Forced expiratory volume in 1 sec<br>Forced vital capacity<br>Height<br>Waist circumference<br>Whole body fat-free mass<br>Whole body water mass
rs2270894	Basal metabolic rate<br>Birth weight<br>Forced vital capacity<br>Height<br>Hip circumference<br>Weight<br>Whole body fat-free mass<br>Whole body water mass
rs2581830	Arm impedance<br>Birth weight<br>BMI<br>Crohns disease<br>Forced vital capacity<br>Hand grip strength<br>Height<br>Hip circumference<br>Trunk fat-free mass<br>Usual walking pace

rs9833926	Basal metabolic rate<br>Hayfever, allergic rhinitis or eczema<br>Heel bone mineral density<br>Height<br>Hematocrit<br>Hemoglobin concentration<br>Hip circumference<br>Lymphocyte count<br>No blood clot, bronchitis, emphysema, asthma, rhinitis, eczema or allergy diagnosed by doctor<br>Red blood cell count<br>Waist circumference<br>Weight<br>Whole body fat-free mass<br>Whole body water mass
rs6439168	Basal metabolic rate<br>Forced expiratory volume in 1 sec<br>Forced vital capacity<br>Height<br>Hip circumference<br>Usual walking pace<br>Weight<br>Whole body fat-free mass<br>Whole body water mass
	Allergic disease<br>Basal metabolic rate<br>Birth weight of first child<br>Body fat percentage<br>Crohns disease

rs724016	Diastolic blood pressure<br>Ever had prostate specific antigen test<br>Forced expiratory volume in 1 sec<br>Forced vital capacity<br>Hair or balding pattern<br>Hand grip strength<br>Hayfever, allergic rhinitis or eczema<br>Heel bone mineral density<br>Height<br>Hip circumference<br>Infant length<br>Lymphocyte count<br>Lymphocyte percentage of white cells<br>Mean corpuscular hemoglobin<br>Mean corpuscular volume<br>Myopia<br>Neutrophil percentage of white cells<br>No blood clot, bronchitis, emphysema, asthma, rhinitis, eczema or allergy diagnosed by doctor<br>Potassium in urine<br>Pulse rate<br>Red blood cell count<br>Relative age of first facial hair<br>Self-reported enlarged prostate<br>Trunk fat percentage<br>Waist circumference<br>Weight<br>Whole body fat mass<br>Whole body fat-free mass

	Whole body water mass
rs13094143	Average weekly beer plus cider intake<br>Basal metabolic rate<br>Height<br>Hip circumference<br>Monokine induced by gamma interferon levels<br>Trunk fat-free mass<br>Whole body fat-free mass<br>Whole body water mass
rs519384	Age at menarche<br>Basal metabolic rate<br>Forced expiratory volume in 1 sec<br>Forced vital capacity<br>Height<br>Impedance<br>Peak expiratory flow<br>Pulse rate<br>Trunk fat-free mass<br>Whole body fat-free mass
rs720390	Basal metabolic rate<br>BMI<br>Diabetes diagnosed by doctor<br>Height<br>Hip circumference<br>Illnesses of father: daibetes<br>Platelet count<br>Plateletcrit<br>Self-reported diabetes

	Waist circumference<br>Whole body fat-free mass<br>Whole body water mass
rs6853216	Age at first live birth<br>Basal metabolic rate<br>Birth weight<br>Body fat percentage<br>Diastolic blood pressure<br>Fluid intellingence score<br>Forced expiratory volume in 1 sec<br>Forced vital capacity<br>Hand grip strength<br>Height<br>Hip circumference<br>Number of treatments or medications taken<br>Peak expiratory flow<br>Peritonsillar abscess<br>Qualifications: A levels or as levels or equivalent<br>Self-reported hypertension<br>Systolic blood pressure<br>Time spent watching tv<br>Vascular or heart problems diagnosed by doctor: high blood pressure<br>Vascular or heart problems diagnosed by doctor: none of the above<br>Waist circumference<br>Weight<br>Whole body fat-free mass<br>Whole body water mass
	Body fat percentage

rs4694504	Height<br>Hip circumference<br>Impedance<br>Waist circumference
rs1975474	Basal metabolic rate<br>Forced expiratory volume in 1 sec<br>Forced vital capacity<br>Height<br>Hip circumference<br>Overall health rating<br>Unspecified haematuria<br>Waist circumference<br>Weight<br>Whole body fat mass<br>Whole body fat-free mass<br>Whole body water mass
rs1562975	Height
rs12648786	Basal metabolic rate<br>Birth weight<br>Forced expiratory volume in 1 sec<br>Forced vital capacity<br>Height<br>Hip circumference<br>Peak expiratory flow<br>Waist hip ratio<br>Weight<br>Whole body fat-free mass<br>Whole body water mass

rs7689420	Basal metabolic rate<br>Forced expiratory volume in 1 sec<br>Forced vital capacity<br>Hand grip strength<br>Height<br>Hip circumference<br>Peak expiratory flow<br>Waist circumference<br>Weight<br>Whole body fat mass<br>Whole body fat-free mass<br>Whole body water mass
rs6450922	Basal metabolic rate<br>Height<br>Hip circumference<br>Whole body fat-free mass<br>Whole body water mass
	Basal metabolic rate<br>Diastolic blood pressure<br>Forced expiratory volume in 1 sec<br>Height<br>Hip circumference<br>Illnesses of siblings: high blood pressure<br>Illnesses of siblings: none of the above, group 1<br>Medication for cholesterol, blood pressure or diabetes: blood pressure medication<br>Medication for cholesterol, blood pressure or diabetes: none of the above<br>Number of self-reported non-cancer illnesses<br>Self-reported gestional hypertension or pre-eclampasia

rs7733331	Self-reported hypertension<br>Systolic blood pressure<br>Treatment with bendroflumethiazide<br>Treatment with blood pressure<br>Treatment with lisinopril<br>Vascular or heart problems diagnosed by doctor: high blood pressure<br>Vascular or heart problems diagnosed by doctor: none of the above<br>Waist circumference<br>Weight<br>Whole body fat mass<br>Whole body fat-free mass<br>Whole body water mass
rs6894139	Basal metabolic rate<br>Bone mineral density<br>Femoral neck bone mineral density<br>Forced expiratory volume in 1 sec<br>Forced vital capacity<br>Forearm bone mineral density<br>Hair or balding pattern<br>Height<br>Platelet count<br>Platelet distribution width<br>Plateletcrit<br>Whole body fat-free mass<br>Whole body water mass
	Asthma<br>Basal metabolic rate<br>Basophil count

rs274567	Blood metabolite levels<br>Crohns disease<br>Eosinophil percentage of granulocytes<br>Eosinophil percentage of white cells<br>Eosinophinil count<br>Granulocyte count<br>Hand grip strength<br>Height<br>High light scatter percentage of red cells<br>Inmature fration of reticulocytes<br>Mean platelet volum<br>Monocyte count<br>Myeloid white cell count<br>Neutrophil count<br>Neutrophil percentage of granulocytes<br>No blood clot, bronchitis, emphysema, asthma, rhinitis, eczema or allergy diagnosed by doctor<br>Platelet count<br>Reticulocyte fraction of red cells<br>Self-reported asthma<br>Self-reported hypertension<br>Sum basophil neutrophil counts<br>Sum eosinophil basophil counts<br>Sum neutrophil eosinophil counts<br>Treatment with bendroflumethiazide<br>Vascular or heart problems diagnosed by doctor: high blood pressure<br>Vascular or heart problems diagnosed by doctor: none of the above<br>Waist circumference<br>Weight

	White blood cell count<br>Whole body fat-free mass<br>Whole body water mass
rs31198	Basal metabolic rate<br>Forced expiratory volume in 1 sec<br>Forced vital capacity<br>Heel bone mineral density<br>Height<br>Whole body fat-free mass<br>Whole body water mass
rs4282339	Basal metabolic rate<br>Forced vital capacity<br>Hand grip strength<br>Height<br>Weight<br>Whole body fat-free mass<br>Whole body water mass
rs153753	Basal metabolic rate<br>Hand grip strength<br>Height<br>Hip circumference<br>Whole body fat-free mass<br>Whole body water mass
rs4868126	Basal metabolic rate<br>Height<br>Hip circumference<br>Waist circumference<br>Weight

	Whole body fat-free mass<br>Whole body water mass
rs4868293	Height<br>Hip circumference
rs2714354	Height
rs3812163	Basal metabolic rate<br>Forced expiratory volume in 1 sec<br>Hand grip strength<br>Height<br>Weight<br>Whole body fat-free mass<br>Whole body water mass
	Basal metabolic rate<br>Birth weight<br>BMI<br>Body fat percentage<br>Breastfed as a baby<br>Disorders mineral metabolism<br>Exposure to tobacco smoke outside home<br>Forced expiratory volume in 1 sec<br>Gene expression<br>Hayfever, allergic rhinitis or eczema<br>Hearing difficulty or problems with background noise<br>Height<br>Hemoglobin concentration<br>High density lipoprotein<br>High light scatter percentage of red cells<br>Hip circumference

rs806794	Mean corpuscular hemoglobin
	Mean corpuscular hemoglobin concentration
	Mean corpuscular volume
	Medication for cholesterol, blood pressure or diabetes: blood pressure medication
	Medication for cholesterol, blood pressure or diabetes: none of the above
	Mouth or teeth dental problems: dentures
	Number of cigarettes previosly smoked daily
	Pack years adult smoking as proportion of life span exposed to smoking
	Pack years of smoking preview only
	Qualifications: A levels or as levels or equivalent
	Qualifications: college or university degree
	Red cell distribution width
	Reticulocyte count
	Reticulocyte fraction of red cells
	Self-reported hypertension
	Time spent watching tv
	Transport type for commuting to job workplace: cycle
	Types of physical activity in last 4 weeks: strenuous sports
	Usual walking pace
	Varicose veins of lower extremities
	Vascular or heart problems diagnosed by doctor: high blood pressure
	Vascular or heart problems diagnosed by doctor: none of the above
	Waist circumference
	Weight
	Whole body fat mass
	Whole body fat-free mass
	Whole body water mass
	Worry too long after embarrassment

	Years educational attainment
rs7744253	Basal metabolic rate
	Basophil count
	Coronary artery disease
	Eosinophinil count
	Forced vital capacity
	Granulocyte count
	Hand grip strength
	Hearing difficulty or problems with background noise
	Height
	Hemoglobin concentration
	Hip circumference
	Intestinal malabsorption
	Lymphocyte count
	Lymphocyte percentage of white cells
	Mean corpuscular hemoglobin concentration
	Mean platelet volum
	Monocyte count
	Mouth or teeth dental problems: mouth ulcers
	Myeloid white cell count
	Neutrophil count
	Peak expiratory flow
	Red cell distribution width
	Rheumatoid arthritis
	Rheumatoid arthritis
	Self-reported ankylosing spondylitis
	Self-reported hypothyroidism or myxoedema
	Self-reported malabsorption or coeliac disease

	Self-reported sarcoidosis<br>Started insulin within one year diagnosis of diabetes<br>Sum basophil neutrophil counts<br>Sum neutrophil eosinophil counts<br>Treatment with levothyroxine sodium<br>Treatment with tyroxine product<br>Weight<br>White blood cell count<br>Whole body fat-free mass<br>Whole body water mass
	Ankylosing spondylitis<br>Basal metabolic rate<br>Basophil count<br>Basophil percentage with white cells<br>Birth weight<br>Diastolic blood pressure<br>Eosinophil percentage of white cells<br>Eosinophinil count<br>Eosiphinil percentage of granulocytes<br>Forced expiratory volume in 1 sec<br>Forced vital capacity<br>Gene expression<br>Granulocyte count<br>Height<br>Hip circumference<br>IgA deficiency<br>Intestinal malabsorption<br>Lymphocyte count

rs2256183	Lymphocyte percentage of white cells<br>Mean platelet volum<br>Medication for pain relief, constipation, heartburn: none of the above<br>Monocyte count<br>Mouth or teeth dental problems: mouth ulcers<br>Myeloid white cell count<br>Neutrophil count<br>Neutrophil percentage of granulocytes<br>Neutrophil percentage of white cells<br>Platelet distribution width<br>Plateletcrit<br>Primary sclerosing cholangitis<br>Red cell distribution width<br>Reticulocyte count<br>Reticulocyte fraction of red cells<br>Rheumatoid arthritis<br>Self-reported adrenocortical insufficiency or addisons disease<br>Self-reported ankylosing spondylitis<br>Self-reported high cholesterol<br>Self-reported hypothyroidism, thyrotocosis or myxoedema<br>Self-reported malabsorption or coeliac disease<br>Self-reported psoriasis<br>Self-reported sarcoidosis<br>Sum basophil neutrophil counts<br>Sum eosinophil basophil counts<br>Total cholesterol<br>Treatment with levothyroxine sodium<br>Unspecified haematuria

	Weight
	White blood cell count
	Whole body water mass
	Basal metabolic rate
	Basophil count
	Diastolic blood pressure
	Doctor diagnosed sarcoidosis
	DPA 22:5n3
	Drive faster than motorway speed limit
	Eosinophinil count
	Eosinophil percentage of granulocytes
	Eosinophil percentage of white cells
	Forced expiratory volume in 1 sec
	Forced vital capacity
	Frequency of tenseness or restlessness in last 2 weeks
	Granulocyte count
	Hand grip strength
	Height
	High light scatter percentage of red cells
	High light scatter reticulocyte count
	Hip circumference
	Impedance
	Intestinal malabsorption
	Lymphocyte count
	Medication for cholesterol, blood pressure or diabetes: cholesterol lowering medication
	Medication for cholesterol, blood pressure or diabetes: none of the above
	Medication for pain relief, constipation, heartburn: none of the above
	Monocyte count

rs6937696	Mouth or teeth dental problems: mouth ulcers<br>Multiple sclerosis<br>Myeloid cell count<br>Neutrophil count<br>Neutrophil percentage of granulocytes<br>Number of self-reported non-cancer illnesses<br>Number of treatments or medications taken<br>Other rheumatoid arthritis<br>Overall health rating<br>Primary sclerosing cholangitis<br>Reticulocyte count<br>Reticulocyte fraction of red cells<br>Rheumatoid arthritis<br>Self-reported adrenocortical insufficiency or addisons disease<br>Self-reported ankylosing spondylitis<br>Self-reported high cholesterol<br>Self-reported hypertension<br>Self-reported hyperthyroidism or thyrotoxicosis<br>Self-reported hypothyroidism or myxoedema<br>Self-reported malabsorption or coeliac disease<br>Self-reported multiple sclerosis<br>Self-reported multiple sclerosis<br>Self-reported psoriasis<br>Self-reported rheumatoid arthritis<br>Self-reported sarcoidosis<br>Self-reported sjogrens syndrome or sicca syndrome<br>Self-reported-systemic lupus erythematosis or sle<br>Seropositive rheumatoid arthritis

	Sum basophil neutrophil counts<br>Sum eosinophil basophil counts<br>Sum neutrophil eosinophil counts<br>Taking other prescription medications<br>Total cholesterol<br>Treatment with baclofen<br>Treatment with carbimazole<br>Treatment with fludrocortisone<br>Treatment with methotrexate<br>Treatment with simvastatin<br>Treatment with sulfasalazine<br>Unspecified haematuria<br>Vascular or heart problems diagnosed by doctor: high blood pressure<br>Vascular or heart problems diagnosed by doctor: none of the above<br>Weight<br>Wheeze or whistling in the chest in last year<br>White blood cell count<br>Whole body fat-free mass<br>Whole body water mass<br>Worrier or anxious feelings
	Arm impedance<br>Basal metabolic rate<br>Birth weight<br>Birth weight of first child<br>Body height<br>BMI<br>Forced expiratory volume in 1 sec<br>Forced vital capacity

rs7742369	Height<br>Hip circumference<br>Home area population density: Scotland large urban area<br>Mean corpuscular volume<br>Peak expiratory flow<br>Platelet count<br>Waist circumference<br>Weight<br>Whole body fat mass<br>Whole body fat-free mass<br>Whole body water mass
rs9462007	Basal metabolic rate<br>Body fat percentage<br>BMI<br>Coronary artery disease<br>Forced expiratory volume in 1 sec<br>HDL cholesterol<br>Height<br>High density lipoprotein<br>Hip circumference<br>Impedance<br>Lymphocyte count<br>Medication for pain relief, constipation, heartburn: none of the above<br>Monocyte count<br>Overall health rating<br>Platelet count<br>Plateletcrit<br>Pulse rate

	Relative age of first facial hair<br>Total cholesterol<br>Treatment with omeprazole<br>Usual walking pace<br>Waist circumference<br>Weight<br>Whole body fat mass<br>Whole body fat-free mass<br>Whole body water mass
rs3734254	Basal metabolic rate<br>Diastolic blood pressure<br>Forced expiratory volume in 1 sec<br>Forced expiratory volume in 1 sec<br>Hand grip strength<br>Height<br>Monocyte percentage of white cells<br>Peak expiratory flow<br>Systolic blood pressure<br>Weight<br>Whole body fat-free mass<br>Whole body water mass
rs9357470	Basal metabolic rate<br>Coxarthrosis<br>Heel bone mineral density<br>Height<br>Hip circumference<br>Whole body fat-free mass<br>Whole body water mass

rs12209223	Basal metabolic rate<br>Forced expiratory volume in 1 sec<br>Height<br>Hip circumference<br>Hip osteoarthritis<br>Waist circumference<br>Weight<br>Whole body fat-free mass<br>Whole body water mass
rs920842	Basal metabolic rate<br>Forced vital capacity<br>Height<br>Weight<br>Whole body fat-free mass<br>Whole body water mass
rs314263	Age at menarche<br>Basal metabolic rate<br>Forced expiratory volume in 1 sec<br>Forced vital capacity<br>Hand grip strength<br>Height<br>Hip circumference<br>Impedance<br>Medication for cholesterol, blood pressure or diabetes: blood pressure medication<br>Medication for pain relief, constipation, heartburn: none of the above<br>Number of operations<br>Number of treatments or medications taken<br>Relative age of first facial hair

	Relative age voice broke
	Sleeplessness or insomnia
	Tanner stage
	Waist circumference
	Weight
	Whole body fat mass
	Whole body fat-free mass
	Whole body water mass
rs9488923 (rs2027850)	Height
rs9388490	Age at menarche
	Age completed full time education
	Basal metabolic rate
	Birth weight
	Body fat percentage
	Coronary artery disease
	Diabetes diagnosed by doctor
	Forced expiratory volume in 1 sec
	Forced vital capacity
	Hair or balding pattern
	Height
	Hematocrit
	Hemoglobin concentration
	Hip circumference
	Impedance
	Intelligence
	Intelligence multi triat analysis
	Intracranial volume
	Nervous feelings

	Pain type experienced in last month: none of the above<br>Red blood cell count<br>Sodium in urine<br>Type II diabetes<br>Weight<br>Whole body fat-free mass<br>Whole body water mass
rs6569648	Basal metabolic rate<br>BMI<br>Breast cancer<br>Breast cancer estrogen receptor negative<br>Creatinine in urine<br>Forced expiratory volume in 1 sec<br>Forced vital capacity<br>Hand grip strength<br>Heel bone mineral density<br>Height<br>Hematocrit<br>Hemoglobin concentration<br>High light scatter reticulocyte count<br>Hip circumference<br>Immature fraction of reticulocytes<br>Impedance<br>Lymphocyte count<br>Maternal effects on offspring birthweight<br>Waist hip ratio<br>Weight<br>White blood cell count

	Whole body fat mass<br>Whole body fat-free mass<br>Whole body water mass
rs9389986	Basal metabolic rate<br>Birth weight<br>Birthlength<br>Forced expiratory volume in 1 sec<br>Forced vital capacity<br>Height<br>Hip circumference<br>Impedance<br>Lung function ratio of forced expiratory volum in 1 sec<br>Waist circumference<br>Weight<br>Whole body fat mass<br>Whole body fat-free mass<br>Whole body water mass
rs827423	Basal metabolic rate<br>Forced expiratory volume in 1 sec<br>Forced vital capacity<br>Heel bone mineral density<br>Height<br>Hip circumference<br>Weight<br>Whole body fat-free mass<br>Whole body water mass
	Arm impedance<br>Basal metabolic rate<br>Birth weight

rs1182199	Body fat percentage<br>Forced expiratory volume in 1 sec<br>Forced vital capacity<br>Height<br>Hip circumference<br>Myeloid white cell count<br>Not blood clot, bronchitis, emphysema, asthma, rhinitis, eczema or allergy diagnosed by doctor<br>Platelet count<br>Plateletcrit<br>Ulcerative colitis<br>Waist circumference<br>Weight<br>White blood cell count<br>Whole body fat mass<br>Whole body fat-free mass<br>Whole body water mass
rs2192476	Height
rs506154	Basal metabolic rate<br>Diabetes diagnosed by doctor<br>Forced expiratory volume in 1 sec<br>Forced vital capacity<br>Height<br>Hip circumference<br>Impedance<br>Self-reported diabetes<br>Treatment with metformin<br>Waist circumference<br>Weight<br>Whole body fat-free mass

	Whole body water mass
rs12673331	Basal metabolic rate<br>Forced expiratory volume in 1 sec<br>Forced vital capacity<br>Height<br>Impedance<br>Whole body fat-free mass<br>Whole body water mass
rs2282978	Basal metabolic rate<br>Birth weight<br>Body height<br>CDK6 expression in lymphocytes<br>Forced expiratory volume in 1 sec<br>Forced vital capacity<br>Granulocyte percentage of myeloid white cells<br>Heel bone mineral density<br>Height<br>Hip circumference<br>Monocyte count<br>Monocyte percentage of white cells<br>Pulse pressure<br>Rheumatoid arthritis<br>Systolic blood pressure<br>Waist circumference<br>Weight<br>Whole body fat mass<br>Whole body fat-free mass<br>Whole body water mass
	Basal metabolic rate<br>Birth weight<br>Forced expiratory volume in 1 sec

rs9650315	Forced vital capacity<br>Height<br>Hip circumference<br>Impedance<br>Weight<br>Whole body fat-free mass<br>Whole body water mass
rs7463906	Basal metabolic rate<br>Forced expiratory volume in 1 sec<br>Forced vital capacity<br>Height<br>Hip circumference<br>Peak expiratory flow<br>Waist circumference<br>Weight<br>Whole body fat-free mass<br>Whole body water mass
rs7463906	Basal metabolic rate<br>Forced expiratory volume in 1 sec<br>Forced vital capacity<br>Height<br>Hip circumference<br>Peak expiratory flow<br>Waist circumference<br>Weight<br>Whole body fat-free mass<br>Whole body water mass
	Basal metabolic rate

rs7463906	Forced expiratory volume in 1 sec<br>Forced vital capacity<br>Height<br>Hip circumference<br>Peak expiratory flow<br>Waist circumference<br>Weight<br>Whole body fat-free mass<br>Whole body water mass
rs7463906	Basal metabolic rate<br>Forced expiratory volume in 1 sec<br>Forced vital capacity<br>Height<br>Hip circumference<br>Peak expiratory flow<br>Waist circumference<br>Weight<br>Whole body fat-free mass<br>Whole body water mass
rs4130415	Basal metabolic rate<br>Forced expiratory volume in 1 sec<br>Forced vital capacity<br>Heel bone mineral density<br>Height<br>Hip circumference<br>Pain type experienced in last month: back pain<br>Peak expiratory flow<br>Self-reported prolapsed disc or slipped disc

	Waist circumference<br>Weight<br>Whole body fat-free mass<br>Whole body water mass
rs4909595	Basal metabolic rate<br>Forced expiratory volume in 1 sec<br>Height<br>Hip circumference<br>Systolic blood pressure<br>Treatment with rennie duo oral suspension<br>Weight<br>Whole body fat-free mass<br>Whole body water mass
rs9407763	Height
rs11144688	Basal metabolic rate<br>Forced expiratory volume in 1 sec<br>Height<br>Hip circumference<br>Impedance<br>Waist circumference<br>Weight<br>Whole body fat-free mass<br>Whole body water mass
	Alcohol usually taken with meals<br>Eosinophil percentage of granulocytes<br>Eosinophil percentage of white cells<br>Granulocyte count<br>Hair or balding pattern

rs7855177	Height<br>Impedance<br>Mean time to correctly identify matches<br>Myeloid white cell count<br>Nap during day<br>Neutrophil count<br>Neutrophil percentage of granulocytes<br>Sum basophil neutrophil counts<br>Sumneutrophil eosinophil<br>White blood cell count
rs3937594	Basal metabolic rate<br>Forced expiratory volume in 1 sec<br>Height<br>Hip circumference<br>Nonrheumatic aortic valve disorders<br>Weight<br>Whole body fat-free mass<br>Whole body water mass
rs7043114	Arm impedance<br>Height
rs4448343	Basal metabolic rate<br>Birth weight<br>Fluid intelligence score<br>Forced expiratory volume in 1 sec<br>Heel bone mineral density<br>Height<br>Hip circumference<br>Time spent using computer

	Waist circumference<br>Whole body fat-free mass<br>Whole body water mass
rs2025151	Basal metabolic rate<br>Forced vital capacity<br>Height<br>Hip circumference<br>Job involves heavy manual or physical work<br>Platelet count<br>Platelet distribution width<br>Pulse rate<br>Time spent watching tv<br>Whole body fat-free mass<br>Whole body water mass
rs4333673	Basal metabolic rate<br>Forced vital capacity<br>Height<br>Whole body fat-free mass<br>Whole body water mass
rs7853634	Basal metabolic rate<br>Forced expiratory volume in 1 sec<br>Forced vital capacity<br>Hand grip strength<br>Height<br>Impedance<br>Weight<br>Whole body fat-free mass<br>Whole body water mass

rs7466269	Basal metabolic rate<br>Bone mineral density paedriatric lower limb<br>Bone mineral density paedriatric total body less head<br>Forced expiratory volume in 1 sec<br>Forced vital capacity<br>Hand grip strength<br>Heel bone mineral density<br>Height<br>Weight<br>Whole body fat-free mass<br>Whole body water mass
rs4980067	Basal metabolic rate<br>Forced vital capacity<br>Height<br>High light scatter percentage of red cells<br>High light scatter reticulocyte count<br>Inmature fraction of reticulocytes<br>Platelet count<br>Reticulocyte count<br>Reticulocyte fraction of red cells<br>Weight<br>Whole body fat-free mass<br>Whole body water mass
rs234886	Basal metabolic rate<br>Height<br>Hip circumference<br>Waist circumference<br>Weight

	Whole body fat-free mass<br>Whole body water mass
rs1879671	Height<br>Treatment with amiodarone
rs606452	Age-related macular degeneration<br>BMI<br>Coronary artery disease<br>Forced expiratory volume in 1 sec<br>Forced vital capacity<br>Height<br>Hip circumference<br>Impedance<br>Waist circumference
rs6487133	Basal metabolic rate<br>Forced expiratory volume in 1 sec<br>Forced vital capacity<br>Height<br>Whole body fat-free mass<br>Whole body water mass
rs11049611	Basal metabolic rate<br>Forced expiratory volume in 1 sec<br>Hand grip strength<br>Height<br>Mouth or teeth dental problems: loose teeth<br>Waist circumference<br>Weight<br>Whole body fat-free mass<br>Whole body water mass

rs1351394	Age at first tooth<br>Alcohol intake frequency<br>Basal metabolic rate<br>Birth length<br>Birth weight<br>BMI<br>Diabetes diagnosed by doctor<br>Estimated total intracranial volume excluding patients with neuropsychiatric disorders<br>Forced expiratory volume in 1 sec<br>Forced vital capacity<br>Hand grip strength<br>Height<br>Hip circumference<br>Impedance<br>Infant head circumference<br>Intracranial volume<br>Maternal effects on offspring birthweight<br>Number of teeth<br>Overall health rating<br>Peak expiratory flow<br>Pulse rate<br>Self-reported diabetes<br>Self-reported high cholesterol<br>Treatment with metformin<br>Waist circumference<br>Weight<br>Whole body fat mass<br>Whole body fat-free mass

	Whole body water mass
rs10748128	Basal metabolic rate<br>Height<br>Hip circumference<br>Waist circumference<br>Weight<br>Whole body fat-free mass<br>Whole body water mass
rs11107116 (rs3825199)	Basal metabolic rate<br>Forced expiratory volume in 1 sec<br>Forced vital capacity<br>Gene expression<br>Heel bone mineral density<br>Height<br>Hip circumference<br>Impedance<br>Standarized difference in height between age 8 years and adult<br>Weight<br>Whole body fat-free mass<br>Whole body water mass
rs11107169	Basal metabolic rate<br>Heel bone mineral density<br>Height<br>Hip circumference<br>Whole body fat-free mass<br>Whole body water mass
	Age at menopause<br>Age completed full time education

rs7980687	Alcohol intake frequency<br>Allergic disease<br>Average weekly red wine intake<br>Basal metabolic rate<br>Brain<br>College completion<br>Diatolic blood pressure<br>Educational attainment<br>Hayfever, allergic rhinitis or eczema<br>Head circumference infant<br>Heel bone mineral density<br>Height<br>Hip circumference<br>Infant head circumference<br>Job involves heavy manual or physical work<br>Lymphocyte count<br>Lymphocyte percentage of white cells<br>Neutrophil percentage of white cells<br>No blood clot, bronchitis, emphysema, asthma, rhinitis, eczema or allergy diagnosed by doctor<br>Platelet count<br>Plateletcrit<br>Qualifications: A levels or as levels or equivalent<br>Qualifications: college or university degree<br>Qualifications: none<br>Qualifications: O levels or GCSEs or equivalent<br>Qualifications: other professional qualifications<br>Schizophrenia<br>Time spent watching tv

	Weight<br>Whole body fat-free mass<br>Whole body water mass<br>Years of education<br>Years of educational attainment
rs7986486	Basal metabolic rate<br>Forced expiratory volume in 1 sec<br>Forced vital capacity<br>Height<br>High light scatter percentage of red cells<br>Impedance<br>Peak expiratory flow<br>Weight<br>Whole body fat-free mass<br>Whole body water mass
rs3118905	Basal metabolic rate<br>Diastolic blood pressure<br>Forced expiratory volume in 1 sec<br>Forced vital capacity<br>Hand grip strength<br>Heel bone mineral density<br>Height<br>Hip circumference<br>Hyperplasia of prostate<br>Impedance<br>Standarized difference in height between age 8 years and adult<br>Systolic blood pressure<br>Weight

	Whole body fat-free mass<br>Whole body water mass
rs1950500	Basal metabolic rate<br>Gene expression<br>Height<br>Weight<br>Whole body fat-free mass<br>Whole body water mass
rs2093210	Age at menarche<br>Basal metabolic rate<br>Eye problems or disorders: glaucoma<br>Forced expiratory volume in 1 sec<br>Forced vital capacity<br>Glaucoma primary open angle glaucoma<br>Heel bone mineral density<br>Height<br>Hip circumference<br>Impedance<br>Self-reported glaucoma<br>Sodium in urine<br>Treatment with xalatan 0.005% eye drops<br>Weight<br>Whole body fat mass<br>Whole body fat-free mass<br>Whole body water mass
	Age at first live birth<br>Age at menarche<br>Basal metabolic rate

rs5742915	Body fat percentage<br>BMI<br>Forced expiratory volume in 1 sec<br>Forced vital capacity<br>Gene expression<br>Hand grip strength<br>Height<br>Hip circumference<br>Number of fays or week of vigorous physical activity 10+ minutes<br>Pagets disease<br>Pulse rate<br>Sleeplessness or insomnia<br>Transport type for commuting to job workplace: cycle<br>Whole body fat-free mass<br>Whole body water mass
rs4483821	Body fat percentage<br>Forced expiratory volume in 1 sec<br>Forced vital capacity<br>Height<br>Hip circumference<br>Impedance<br>Myopia<br>Waist circumference<br>Weight<br>Whole body fat mass
	Arm impedance<br>Body fat percentage<br>Forced expiratory volume in 1 sec

rs2280470	Forced vital capacity<br>Height<br>Hip circumference<br>Infant length<br>Waist circumference<br>Weight<br>Whole body fat mass<br>Whole body fat-free mass<br>Whole body water mass
rs3784757	Body fat percentage<br>Forced expiratory volume in 1 sec<br>Forced vital capacity<br>Height<br>Weight<br>Whole body fat-free mass<br>Whole body water mass
rs2871865	Basal metabolic rate<br>Birth weight<br>Birth weight of first child<br>Forced expiratory volume in 1 sec<br>Forced vital capacity<br>Hand grip strength<br>Height<br>Weight<br>Whole body fat-free mass<br>Whole body water mass
	Forced vital capacity<br>Height

rs4548838	Hip circumference<br>Impedance<br>Waist circumference
rs4548838	Forced expiratory volume in 1 sec<br>Forced vital capacity<br>Height<br>Hip circumference<br>Impedance<br>Waist circumference
rs8057808	Basal metabolic rate<br>Fluid intelligence score<br>Height<br>Hip circumference<br>Impedance<br>Overall health rating<br>Pulse rate<br>Qualifications: college or university degree<br>Qualifications: none<br>Self-reported hypertension<br>Time spent watching tv<br>Time spent watching tv<br>Vascular or heart problems diagnosed by doctor: high blood pressure<br>Weight<br>Whole body fat-free mass<br>Whole body water mass
	Age at menarche<br>Body fat percentage<br>BMI

rs11866219	Forced expiratory volume in 1 sec<br>Forced vital capacity<br>Hand grip strength<br>Height<br>Hip circumference<br>Illness, injury, bereavement, stress in last 2 years: illness, injury or assault to yourself<br>Leg impedance<br>Long-standing illness, disability or infirmity<br>Number of self-reported non-cancer illnesses<br>Pulse rate<br>Usual walking pace<br>Waist circumference<br>Whole body fat mass
rs7213608	Body fat percentage<br>BMI<br>Forced expiratory volume in 1 sec<br>Height<br>High light scatter percentage of red cells<br>Inmature fraction of reticulocytes<br>Mean corpuscular volume<br>Qualifications: A levels or as levels or equivalent<br>Treatment with simvastatin<br>Waist circumference<br>Whole body fat-free mass<br>Whole body water mass
	Basal metabolic rate<br>BMI<br>Forced expiratory volume in 1 sec

rs3764419	Gene expression<br>Granulocyte percentage of myeloid white cells<br>Height<br>Hip circumference<br>Mean corpuscular hemoglobin<br>Mean corpuscular volume<br>Neutrophil percentage of white cells<br>Waist circumference<br>Weight<br>Whole body fat-free mass<br>Whole body water mass
rs584828	Basal metabolic rate<br>Forced vital capacity<br>Granulocyte count<br>Height<br>Hip circumference<br>Neutrophil count<br>Self-reported thyroid radioablation therapy<br>Sum basophil neutrophil counts<br>Sum neutrophil eosinophil counts<br>Weight<br>White blood cell count<br>Whole body fat-free mass<br>Whole body water mass
[illegible]	Allergic disease<br>Basal metabolic rate<br>Hair or balding pattern<br>Hand grip strength<br>Height<br>Impedance<br>No blood clot, bronchitis, emphysema, asthma, rhinitis, eczema or allergy diagnosed by doctor

rs11657325	Overall health rating
	Sleeplessness or insomnia
	Systolic blood pressure
	Vascular or heart problems diagnosed by doctor: none of the above
	Weight
	Whole body fat-free mass
	Whole body water mass
rs12453394	Basal metabolic rate
	Coronary artery disease
	Drive faster than motorway speed limit
	Forced vital capacity
	Frequency of tiredness or lethargy in last 2 weeks
	Gene expression of UBE2Z in blood
	Height
	Illness of mother: high blood pressure
	Illness of mother: none of the above
	Type II diabetes
	Whole body fat-free mass
	Whole body water mass
rs963292	Arm fat-free mass
	Forced expiratory volume in 1 sec
	Height
	Trunk fat-free mass
rs2079795 (rs7214743)	Gene expression
	Height
	Waist circumference
	Waist hip ratio
	Height

rs2727317	Hip circumference<br>Waist hip ratio
rs4239437	Acute pharyngitis<br>Basal metabolic rate<br>Forced expiratory volume in 1 sec<br>Hand grip strength<br>Heel bone mineral density<br>Height<br>Hip circumference<br>Mean platelet volume<br>Platelet count<br>Platelet distribution width<br>Waist circumference<br>Waist hip ratio<br>Weight<br>Whole body fat mass<br>Whole body fat-free mass<br>Whole body water mass
rs9967417	Basal metabolic rate<br>Forced expiratory volume in 1 sec<br>Gene expression<br>Hand grip strength<br>Height<br>Hip circumference<br>Weight<br>Whole body fat-free mass<br>Whole body water mass
	Basal metabolic rate

rs12980348	Forced expiratory volume in 1 sec<br>Hand grip strength<br>Heel bone mineral density<br>Height<br>Hematocrit<br>Hemoglobin concentration<br>Hip circumference<br>Red blood cell count<br>Weight<br>Whole body fat-free mass<br>Whole body water mass
rs891088	Basal metabolic rate<br>Height<br>Hip circumference<br>Serum urate<br>Weight<br>Whole body fat-free mass<br>Whole body water mass
	Basal metabolic rate<br>Blood clot in the lung<br>Coronary artery disease<br>Forced expiratory volume in 1 sec<br>Forced vital capacity<br>Height<br>High light scatter percentage of red cells<br>High light scatter reticulocyte count<br>Hip circumference<br>Inmature fraction of reticulocytes<br>Low density lipoprotein

rs2738039 (rs1982075)	Morning or evening person<br>Pain type experienced in last month: knee pain<br>Peak expiratory flow<br>Platelet count<br>Plateletcrit<br>Red cell distribution width<br>Reticulocyte count<br>Reticulocyte fraction of red cells<br>Self-reported osteoarthritis<br>Self-reported pulmonary embolism + or - dvt<br>Time spent watching tv<br>Total cholesterol<br>Weight<br>Whole body fat-free mass<br>Whole body water mass
rs6038566	Basal metabolic rate<br>BMI<br>Forced expiratory volume in 1 sec<br>Height<br>Hip circumference<br>Impedance<br>Waist circumference<br>Whole body fat-free mass<br>Whole body water mass
	Basal metabolic rate<br>Forced expiratory volume in 1 sec<br>Forced vital capacity<br>Heel bone mineral density

rs7274811	Height<br>Hip circumference<br>Impedance<br>Weight<br>Whole body fat mass<br>Whole body fat-free mass<br>Whole body water mass
rs6142159	Basal metabolic rate<br>Forced expiratory volume in 1 sec<br>Hair or balding pattern<br>Hand grip strength<br>Heel bone mineral density<br>Height<br>High density lipoprotein<br>Impedance<br>log eGFR creatinine<br>Past tobacco smoking<br>Peak expiratory flow<br>Weight<br>Whole body fat-free mass<br>Whole body water mass
	Acquired deformities of fingers and toes<br>Basal metabolic rate<br>Birth weight<br>Forced expiratory volume in 1 sec<br>Forced vital capacity<br>Gene expression<br>Gonarthrosis

rs143384	Hand grip strength<br>Height<br>Hip circumference<br>Impedance<br>Infant length<br>Internal derangement of knee<br>Joint mobility Beighton score<br>Maximum heart rate during fitness test<br>Pain type experienced in last month: knee pain<br>Peak expiratory flow<br>Qualifications: A levels or as levels or equivalent<br>Relative age voice broke<br>Total cholesterol<br>Usual walking pace<br>Waist circumference<br>Waist hip ratio<br>Weight<br>Whole body fat mass<br>Whole body fat-free mass<br>Whole body water mass
rs4821083 (rs2413143)	Advanced age related macular degeneration<br>Advanced age related macular degeneration choroidal neovascularization vs no AMD<br>Arm fat-free mass<br>Height<br>Hip circumference<br>Impedance<br>Neovascularization<br>Trunk fat-free mass
	Guilty feelings

rs738288	Height<br>IGP40<br>Worry too long after embarrassment

PC3	
**SNP (lead SNP)**	**Trait**
rs7536458	Basal metabolic rate<br>Forced expiratory volume in 1 sec<br>Hair or balding pattern<br>Height<br>Hip circumference<br>Infant length<br>Leg impedance<br>Waist circumference<br>Waist hip ratio<br>Whole body fat-free mass<br>Whole body water mass
rs10923724	BMI<br>Hair or balding pattern<br>Height<br>Impedance<br>Relative age of first facial hair<br>Waist circumference<br>Waist hip ratio
rs9425301	Height<br>Hip circumference<br>Inmature fraction of reticulocytes<br>Mean platelet volume<br>Platelet distribution width<br>Waist circumference<br>Waist hip ratio

	Whole body water mass
rs2274432	Basal metabolic rate<br>Forced expiratory volume in 1 sec<br>Forced vital capacity<br>Hand grip strength<br>Height<br>Hip circumference<br>Leg impedance<br>Myopia<br>Waist circumference<br>Weight<br>Whole body fat mass<br>Whole body fat-free mass<br>Whole body water mass
rs2791550 (rs2605108)	Body fat percentage<br>Falls in the last year<br>Hip circumference<br>Impedance<br>log Fasting insulin<br>Type II diabetes<br>Umbilical hernia<br>Waist circumference<br>Waist hip ratio<br>Whole body fat mass
	Age at menarche<br>Body fat percentage<br>BMI<br>Childhood BMI

rs6726261	Childhood obesity<br>Forced expiratory volume in 1 sec<br>Forced vital capacity<br>Hand grip strength<br>Height<br>Hip circumference<br>Impedance<br>Obesity class 1<br>Obesity with early age of onset age 2<br>Standarized difference in height between age 8 years and adult<br>Waist circumference<br>Weight<br>Whole body fat mass
rs2289195	Basal metabolic rate<br>Forced expiratory volume in 1 sec<br>Forced vital capacity<br>Hand grip strength<br>Height<br>Leg impedance<br>Waist circumference<br>Whole body fat-free mass<br>Whole body water mass
rs6715793	Forced expiratory volume in 1 sec<br>Height<br>Impedance<br>Waist circumference
	Basal metabolic rate<br>Forced expiratory volume in 1 sec

rs3791675	Forced vital capacity<br>Height<br>Hip circumference<br>Impedance<br>Inguinal hernia<br>Mononeuropathies of upper limb<br>Peak expiratory flow<br>Waist circumference<br>Weight<br>Whole body fat mass<br>Whole body fat-free mass<br>Whole body water mass
rs1128249	Body fat percentage<br>BMI<br>Diabetes diagnosed by doctor<br>HDL cholesterol<br>Hemoglobin concentration<br>High density lipoprotein<br>High light scatter percentage of red cells<br>High light scatter reticulocyte percentage of red cells<br>Hip circumference<br>Illness of mother: diabetes<br>Illness of siblings: diabetes<br>Impedance<br>LDL cholesterol<br>log Fasting insuline<br>Low density lipoprotein<br>Reticulocyte count

	Self-reported diabetes<br>Total cholesterol<br>Treatment with metformin<br>Triglycerides<br>Type II diabetes<br>Waist circumference<br>Waist hip ratio<br>Weight<br>Whole body fat mass
rs2342296	Basal metabolic rate<br>Forced expiratory volume in 1 sec<br>Height<br>Waist circumference<br>Whole body fat-free mass<br>Whole body water mass
rs2920499	Height<br>Lymphocyte count<br>Mean corpuscular hemoglobin concentration<br>Mean corpuscular volume<br>Platelet count<br>Plateletcrit<br>Red cell distribution width<br>Total cholesterol<br>Waist hip ratio
	Adiponectin levels<br>Age at menarche<br>Bipolar disorder<br>BMI

rs2710323	Cross disorder<br>Glaucome primary open angle<br>Hematocrit<br>Hemoglobin concentration<br>High light scatter reticulocyte percentage of red cells<br>High light scatter reticulocyte count<br>Hip circumference<br>Hip osteoarthritis<br>Impedance<br>log Adiponectin<br>Nervous feelings<br>Pulse rate<br>Red blood cell count<br>Red cell distribution width<br>Reticulocyte count<br>Reticulocyte fraction of red count<br>Schizophrenia<br>Schizophrenia schizoaffective disorder or bipolar disorder<br>Waist circumference<br>Waist hip ratio<br>Whole body water mass<br>Worrier or anxious feeling
rs9864077	Advanced age related macular degeneration<br>Age-related macular degeneration<br>Arm impedance<br>Body fat percentage<br>BMI<br>Hip circumference

	Type II diabetes<br>Waist circumference<br>Waist hip ratio<br>Whole body fat mass
rs9838625	Allergic disease<br>Basal metabolic rate<br>Forced expiratory volume in 1 sec<br>Forced vital capacity<br>Hair or balding pattern<br>Hayfever, allergic rhinitis or eczema<br>Height<br>Hip circumference<br>Lymphocyte count<br>Mean corpuscular hemoglobin<br>Mean corpuscular volume<br>Myopia<br>Pulse rate<br>Red blood cell count<br>Waist circumference<br>Weight<br>Whole body fat mass<br>Whole body fat-free mass<br>Whole body water mass
rs1545854	Birth weight<br>Heel bone mineral density<br>Height<br>Leg impedance
	Age at first live birth

rs4057984	Basal metabolic rate<br>Birth weight<br>Body fat percentage<br>Diastolic blood pressure<br>Fluid intelligence score<br>Forced expiratory volume in 1 sec<br>Forced vital capacity<br>Hand grip strength<br>Height<br>Hip circumference<br>Number of treatments or medications taken<br>Peak expiratory flow<br>Peritonsillar abscess<br>Qualifications: A levels or as levels or equivalent<br>Qualifications: college or university degree<br>Self-reported hypertension<br>Systolic blood pressure<br>Time spent watching tv<br>Vascular or heart problems diagnosed by doctor: high blood pressure<br>Vascular or heart problems diagnosed by doctor: none of the above<br>Waist circumference<br>Weight<br>Whole body fat-free mass<br>Whole body water mass
	Basal metabolic rate<br>Forced expiratory volume in 1 sec<br>Height<br>Hip circumference

rs1115919	Leg impedance<br>Overall health rating<br>Standarized difference in height between age 8 years and adult<br>Waist circumference<br>Weight<br>Whole body fat mass<br>Whole body fat-free mass<br>Whole body water mass
rs7689420	Basal metabolic rate<br>Forced expiratory volume in 1 sec<br>Forced vital capacity<br>Hand grip strength<br>Height<br>Hip circumference<br>Peak expiratory flow<br>Waist circumference<br>Weight<br>Whole body fat mass<br>Whole body fat-free mass<br>Whole body water mass
	Asthma<br>Atopic dermatitis<br>Basal metabolic rate<br>Basophil count<br>Eosinophil count<br>Eosinophil percentage of granulocytes<br>Eosinophil percentage of white cells<br>Granulocyte count

rs272885	Hand grip strength<br>Height<br>High light scatter percentage of red cells<br>High light scatter reticulocyte count<br>Inmature fraction of reticulocytes<br>Mean platelet volume<br>Monocyte count<br>Myeloid white cell count<br>Neutrophil count<br>Neutrophil percentage of granulocytes<br>No blood, clot, bronchitis, emphysema, asthma, rhinitis, eczema or allergy diagnosed by doctor<br>Platelet count<br>Self-reported asthma<br>Self-reported hypertension<br>Sum basophil neutrophil counts<br>Sum eosinophil basophil counts<br>Sum neutrophil eosinophil counts<br>Time spent using computer<br>Vascular or heart problems diagnosed by doctor: high blood pressure<br>Waist circumference<br>White blood cell count<br>Whole body fat-free mass<br>Whole body water mass
rs6556301	Activated partial thromboplastin time<br>Height<br>Impedance<br>Waist circumference<br>Waist hip ratio

rs6556301	Activated partial thromboplastin time<br>Height<br>Impedance<br>Waist circumference<br>Waist hip ratio
rs1294421	Hip circumference<br>Inguinal hernia<br>Waist circumference<br>Waist hip ratio
rs1776897	Basal metabolic rate<br>Birth weight<br>Birth weight of first child<br>BMI<br>Forced expiratory volume in 1 sec<br>Forced vital capacity<br>Height<br>Hip circumference<br>Peak expiratory flow<br>Platelet count<br>Plateletcrit<br>Waist circumference<br>Waist hip ratio<br>Weight<br>Whole body fat mass<br>Whole body fat-free mass<br>Whole body water mass
	Age at menarche<br>Basal metabolic rate

rs7759938	Forced expiratory volume in 1 sec<br>Forced vital capacity<br>Hand grip strength<br>Height<br>Hip circumference<br>Impedance<br>log Fasting insulin<br>Medication for cholesterol, blood pressure or diabetes: blood pressure medication<br>Medication for pain relief, constipation, heartburn: none of the above<br>Menarche<br>Menarche age at onset<br>Number of operations<br>Number of treatments or medications taken<br>Pubertal anthropometrics<br>Pubertal height growth<br>Relative age voice broke<br>Sleeplessness or insomnia<br>Standarized difference in height between age 14 years and adult<br>Tanner stage<br>Waist circumference<br>Weight<br>Whole body fat mass<br>Whole body fat-free mass<br>Whole body water mass
	Fracture of forearm<br>Fractured bone site: wrist<br>Fractured or broken bones in last 5 years<br>Heel bone mineral density

rs1936809	Hematocrit<br>Hemoglobin concentration<br>High density lipoprotein<br>High light scatter reticulocyte count<br>Hip circumference<br>Lymphocyte count<br>Other orthopaedic follow-up care<br>Red blood cell count<br>Reticulocyte count<br>Triglycerides<br>Varicose veins of lower extremities<br>Waist circumference<br>Waist hip ratio
rs7798002	Basal metabolic rate<br>Height<br>Waist circumference<br>Waist hip ratio<br>Whole body fat-free mass<br>Whole body water mass
rs7801581	Height<br>Waist circumference<br>Waist hip ratio
rs1516883	Age at menarche<br>BMI<br>Forced expiratory volume in 1 sec<br>Forced vital capacity<br>Height<br>Impedance

	Relative age of first facial hair<br>Relative age voice broke<br>Standarized difference in height between age 14 years and adult<br>Waist circumference
rs7021911	Basal metabolic rate<br>Femoral neck bone mineral density<br>Forced expiratory volume in 1 sec<br>Heel bone mineral density<br>Height<br>Whole body fat-free mass<br>Whole body water mass
rs1628023	Basal metabolic rate<br>Diabetes diagnosed by doctor<br>Height<br>Monocyte percentage of white cells<br>Self-reported diabetes<br>Waist circumference<br>Weight<br>Whole body fat-free mass<br>Whole body water mass
	Allergic disease<br>Basophil count<br>Diastolic blood pressure<br>Granulocyte count<br>Hayfever, allergic rhinitis or eczema<br>Height<br>High light scatter percentage of red cells<br>High light scatter reticulocyte count

rs7917772	Leg impedance<br>Mean corpuscular hemoglobin<br>Mean corpuscular hemoglobin<br>Myeloid white cell count<br>Neutrophil count<br>No blood, clot, bronchitis, emphysema, asthma, rhinitis, eczema or allergy diagnosed by doctor<br>Past tobacco smoking<br>Reticulocyte fraction of red cells<br>Sum basophil neutrophil counts<br>Sum neutrophil eosinophil counts<br>Sum eosinophil basophil counts<br>Waist circumference<br>Waist hip ratio<br>White blood cell count
	Age at menarche<br>Alcohol intake frequency<br>Birth weight<br>Body fat percentage<br>BMI<br>Current tobacco smoking<br>Fluid intelligence score<br>Forced expiratory volume in 1 sec<br>Forced vital capacity<br>Frequency of depressed mood in last 2 weeks<br>Gene expression<br>Granulocyte count<br>Granulocyte percentage of myeloid white cells<br>Hand grip strength

rs1317149 (rs12787112)	HDL cholesterol<br>Height<br>High density lipoprotein<br>Hip circumference<br>Immature fraction of reticulocytes<br>log Proinsulin<br>Loneliness, isolation<br>Miserableness<br>Monocyte percentage of white cells<br>Mood swings<br>Neuroticism<br>Neutrophil count<br>Platelet count<br>Plateletcrit<br>Sleeplessness or insomnia<br>Smoking status: current<br>Suffer from nerves<br>Sum basophil neutrophil counts<br>Sum neutrophil eosinophil counts<br>Total cholesterol<br>Usual walking pace<br>Waist circumference<br>White blood cell count<br>Whole body fat mass
rs7949030	Forced expiratory volume in 1 sec<br>Forced vital capacity<br>Impedance<br>Pulse rate

	Waist hip ratio
rs8756	Alcohol intake frequency
	Basal metabolic rate
	Birth weight
	Birth weight of first child
	Birthlength
	BMI
	Diabetes diagnosed by doctor
	Estimated total intracranial volume excluding patients with neuropsychiatric disorders
	Forced expiratory volume in 1 sec
	Forced vital capacity
	Hand grip strength
	Height
	Hip circumference
	Impedance
	Infant head circumference
	Intracranial volume
	Maternal effects on offspring birthweight
	Overall health rating
	Peak expiratory flow
	Pulse rate
	Self-reported diabetes
	Self-reported high cholesterol
	Systolic blood pressure
	Treatment with metformin
	Waist circumference
	Weight
	Whole body fat mass

	Whole body fat-free mass<br>Whole body water mass
rs12303671	Adiponectin levels<br>Body fat percentage<br>BMI<br>Hand grip strength<br>HDL cholesterol<br>Heel bone mineral density<br>High density lipoprotein<br>High light scatter reticulocyte count<br>Hip circumference<br>Impedance<br>log Adiponectin<br>Lymphocyte count<br>Reticulocyte count<br>Reticulocyte fraction of red cells<br>Triglycerides<br>Waist circumference<br>Waist hip ratio<br>White blood cell count<br>Whole body fat mass
rs7492628	Basal metabolic rate<br>BMI<br>Hip circumference<br>Impedance<br>Pulse rate<br>Waist hip ratio<br>Weight

	Whole body fat mass<br>Whole body fat-free mass<br>Whole body water mass
rs16976826 (rs12443429)	Waist circumference<br>Waist hip ratio
rs12440667	Exfoliation glaucoma<br>Forced expiratory volume in 1 sec<br>Forced vital capacity<br>Hand grip strength<br>Height<br>Impedance<br>Peak expiratory flow<br>Waist circumference
rs6602997	Body fat percentage<br>Forced expiratory volume in 1 sec<br>Forced vital capacity<br>Height<br>Hip circumference<br>Impedance<br>Waist circumference<br>Weight<br>Whole body fat mass
rs2280470	Arm impedance<br>Basal metabolic rate<br>Forced expiratory volume in 1 sec<br>Forced vital capacity<br>Height<br>Hip circumference

[illegible]	Infant length
	Waist circumference
	Weight
	Whole body fat mass
	Whole body fat-free mass
	Whole body water mass
	Forced expiratory volume in 1 sec
	Hair or balding pattern
rs1879143	Height
	Peak expiratory flow
	Trunk fat-free mass
	Waist circumference
	Basal metabolic rate
	Forced expiratory volume in 1 sec
	Gene expression
	Height
rs9892365	Systolic blood pressure
	Waist circumference
	Weight
	Whole body fat-free mass
	Whole body water mass
	Acute pharyngitis
	Basal metabolic rate
	Forced expiratory volume in 1 sec
	Hand grip strength
	Heel bone mineral density
	Height
	Hip circumference

rs4239436	Mean platelet volume<br>Platelet count<br>Platelet distribution width<br>Waist circumference<br>Waist hip ratio<br>Weight<br>Whole body fat mass<br>Whole body fat-free mass<br>Whole body water mass
rs6140050	BMI<br>Forced expiratory volume in 1 sec<br>Heel bone mineral density<br>Height<br>High light scatter reticulocyte count<br>Impedance<br>Lung function FVC<br>Platelet count<br>Plateletcrit<br>Snoring<br>Waist circumference<br>Waist hip ratio<br>Whole body fat-free mass<br>Whole body water mass
rs2179129	Arm impedance<br>Diastolic blood pressure<br>Hair or balding pattern<br>Systolic blood pressure<br>Vascular or heart problems diagnosed by doctor: high blood pressure<br>Waist circumference<br>Waist hip ratio
